# Surpassing kilometer-scale terahertz wireless communication beyond 300 GHz enabled by hybrid photonic–electronic synergy

**DOI:** 10.1038/s41377-026-02321-6

**Published:** 2026-05-09

**Authors:** Yuancheng Cai, Lin Zhang, Jiao Zhang, Bingchang Hua, Kexin Ma, Junjie Ding, Xingwang Bian, Mingzheng Lei, Yingzhou Liu, Jiankang Li, Zhigang Xin, Xingyu Chen, Jun Cai, Pan Pan, Yongming Huang, Jinjun Feng, Min Zhu, Xiaohu You

**Affiliations:** 1 Purple Mountain Laboratories, Nanjing, China; 2 National Mobile Communications Research Laboratory, Southeast University, Nanjing, China; 3 National Key Laboratory of Science and Technology on Vacuum Electronics, Beijing Vacuum Electronics Research Institute, Beijing, China

**Keywords:** Terahertz optics, Terahertz optics, Microwave photonics, Fibre optics and optical communications

## Abstract

Terahertz (THz) bands are critical for next-generation wireless fronthaul/backhaul applications. However, they face a fundamental coverage range limitation due to low emission power, severe path loss, and poor receiving sensitivity, especially in photonics-assisted THz systems beyond 300 GHz. To address this limitation, we develop a 335 GHz continuous-wave traveling wave tube amplifier with an output power close to 4 W and a gain of over 50 dB, and construct a novel yet simple diversity receiving scheme to improve the receiving signal-to-noise ratio by ~3 dB. Through hybrid photonic–electronic synergy, combining photonics-assisted THz generation, high-power THz amplification, and spatial diversity reception, a record-breaking kilometer-scale THz wireless communication at 335 GHz—a highly challenging atmospheric window—is demonstrated. We first achieve a net rate of 27.84 Gbit s^−1^ over a 2.2 km wireless link—yielding an unprecedented rate–distance product of 61,248 Gbit s^−1^ ∙ m—beyond 300 GHz to the best of our knowledge.

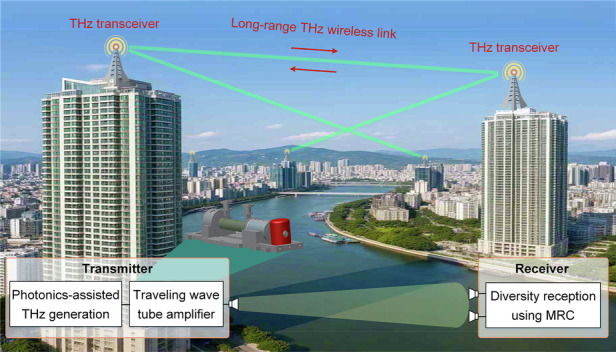

## Abstract (web summary)

A hybrid photonic–electronic synergy integrates photonics-assisted THz generation, high-power traveling wave tube amplification, and diversity reception, enabling kilometer-scale 335 GHz wireless communication with a record 61,248 Gbit s^-1^·m rate–distance product.

## Introduction

**Fig. 1:**
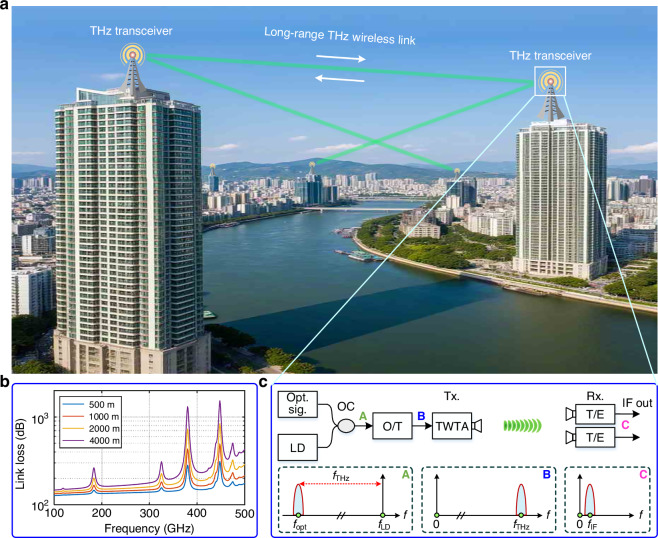
Point-to-point long-range THz wireless communication based on a photonic–electronic converged solution. This work focuses on photonics-assisted THz signal generation with high-gain amplification at the transmitter and high-sensitivity THz reception at the receiver, enabling high-speed and long-range THz wireless communication. **a** Vision of a point-to-point long-range THz wireless link. In certain scenarios where optical fiber deployment is inconvenient or extremely expensive, high-speed THz wireless links can replace optical fibers to reduce overall deployment costs. **b** Air propagation loss of THz waves for standard conditions (temperature, 25 °C; relative humidity, 40%; standard atmospheric pressure). The total link loss of THz waves above 300 GHz exceeds 150 dB. **c** Concept of a THz transceiver based on a photonic–electronic converged solution. The photonics-assisted approach is used for high-frequency and high-speed THz signal generation. One high-gain TWTA is used to amplify the power of the resultant THz signal before emission, enough to overcome high path losses. Insets A and B show the corresponding optical and electrical spectra before and after O/T, respectively. Inset C shows the electrical spectrum after T/E. LD laser diode, OC optical coupler, O/T optical-to-THz conversion, T/E THz-to-electric conversion, Tx transmitter, Rx receiver, TWTA traveling wave tube amplifier, IF intermediate frequency

The explosive growth of data traffic in wireless communications has driven the exploration of unoccupied high-frequency terahertz (THz) bands above 300 GHz, offering large bandwidths to meet the demand for tens or even hundreds of gigabits per second of data rate^1–3^. Such high-speed THz wireless links are critical for wireless fronthaul/backhaul^4,5^ networks and emergency communications, especially in scenarios where optical fiber deployment is inconvenient or extremely expensive, such as across mountains, rivers, and remote areas (Fig. 1a). In this context, overall deployment costs can be significantly reduced. In the last decade, the photonics-assisted THz communication technique has emerged as a particularly promising approach^6–8^. It not only easily achieves high-frequency^9–12^, high-speed^13,14^, and real-time^15,16^ THz communications but also enables the seamless integration of THz wireless links into mature optical fiber infrastructures^17–19^.

However, the wireless transmission distance of high-frequency THz communications enabled by photonics is extremely limited, mainly because of the following two reasons. On the one hand, intrinsic propagation loss, including free space path loss and atmospheric absorption loss of high-frequency THz waves, especially for carrier frequencies above 300 GHz, can reach tens of decibels per kilometer or more^7,20^. As shown in Fig. 1b, the overall loss of a 300 GHz THz wave after 1 km atmospheric transmission is over 150 dB under standard environment conditions. On the other hand, the optical-to-THz (O/T) conversion, usually using positive-intrinsic-negative photodiodes^21^ or uni-traveling carrier photodiodes (UTC-PD)^22,23^, only has a power conversion efficiency of less than 2% in the 300 GHz band and above. As a result, the output power of the THz signal generated by heterodyne photomixing between two lightwaves is often limited to the microwatt level. Therefore, at present, the expansion of THz wireless transmission distance has to rely on key amplification components. Unfortunately, although state-of-the-art solid-state THz amplifiers above 300 GHz can increase the THz emission power to some extent, long-range THz wireless transmission toward the kilometer level for photonics-assisted THz communications still faces great challenges, limited by low amplification gains and low saturation output power.

In this paper, we demonstrate a photonic–electronic converged THz long-range wireless transmission link above 300 GHz, employing a self-designed high-power and high-gain THz transmitting component and a high-sensitivity THz receiving architecture. The wireless link transmits at line rates of up to 34.8 Gbit s^−1^ and operates at a THz carrier frequency of 335 GHz over a record-breaking wireless distance of 2.2 km, presenting an unprecedented rate–distance product above the 300 GHz band for the first time to our knowledge. The high emission THz power at 335 GHz carrier frequency is derived from our self-developed continuous-wave traveling wave tube amplifier (TWTA) module. This TWTA module is designed with a transformative folded waveguide (FWG) slow-wave structure (SWS), which enables a continuous output power up to 3.82 W and a signal gain of over 50 dB. Figure 1c shows the concept of the THz transceiver based on a photonic–electronic converged solution. The photonics-assisted THz generation approach based on O/T conversion is used to generate high-frequency and high-rate THz signals, and the high-gain continuous-wave TWTA is used to amplify the THz signal power enough to counteract the link losses of long-range THz wireless transmission. Additionally, the diversity receiving scheme based on a single-emission and double-reception wireless transmission architecture is employed to improve the THz receiving sensitivity. We expect that the photonic–electronic converged THz communication solution based on the combination of wideband O/T conversion with high-power THz amplification in a compact form can greatly accelerate the development of long-range THz communications and advance the integration of THz wireless links into mature optical fiber infrastructures.

## Results

### Design of a 335 GHz continuous-wave TWTA

Among various THz amplifiers, vacuum-electronics-based TWTAs have emerged as the most promising high-power THz amplification components given their distinctive features, including their high power capacity, high signal gain, and high reliability^24^. Notably, TWTAs above 300 GHz have achieved watt-level output power^25,26^, and the simulated output power for TWTA below 300 GHz can even approach a hundred-watt level^27^, significantly outperforming solid-state power amplifiers (SSPAs) by over an order of magnitude at comparable frequencies^28^. This breakthrough underscores their potential in addressing the escalating demand for high-power THz communication, sensing, and imaging fields, especially for the photonics-assisted THz technical route.

**Fig. 2:**
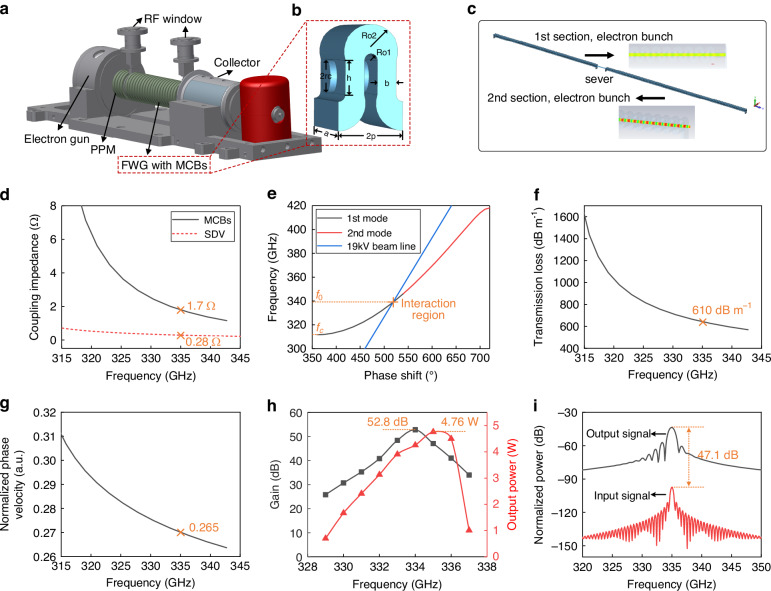
Design of a 335 GHz continuous-wave TWTA. **a** Assembly drawing of the TWTA module. **b** Enlarged diagram of SWS; MCB SWS is used in this TWTA. **c** A two-section MCB model in CST Particle Studio. Different sections have different effects on electron bunching. **d** Coupling impedance comparison between SDV and MCBs. **e** Brillouin zone diagram of the MCBs with a beam line of 19 kV. The first and second modes are the electric field distributions of the first and second eigenmodes, respectively, with both corresponding to the TE_10_ mode. **f** Transmission loss of MCBs. **g** Normalized phase velocity of MCBs. **h** Simulated output power and gain of the 335 GHz TWTA. **i** Frequency spectrum at 335 GHz. SWS slow-wave structure, PPM periodic permanent magnet, FWG folded waveguide, MCBs modified circular bends, SDV staggered double vane

TWTAs operate by amplifying signals through dynamic energy exchange between the electron beam and guided electromagnetic waves. This interaction is enabled by five core components—Pierce electron gun, periodic permanent magnet (PPM), FWG SWS, low-loss radio frequency (RF) window, and multistage depressed collector (Fig. 2a). However, the scaling of TWTAs to THz frequencies faces critical challenges stemming from fundamental physical limitations. The core of these challenges is severe attenuation loss in the SWS. It degrades energy transfer efficiency between the electron beam and electromagnetic waves, directly limiting both gain and output power^24^. Furthermore, compounding this issue is the miniaturization constraint. As critical dimensions shrink to the micrometer scale, even minor misalignments in the electron beam trajectory dramatically reduce transmission efficiency. This not only destabilizes beam–wave interaction but also hinders continuous-wave operation because of beam scattering in the microscale and channels excessive thermal loading. However, continuous-wave operation is one of the fundamental requirements for THz communications.

Although state-of-the-art TWTA prototypes over 300 GHz deliver 1 W output power with 18 dB gain, these metrics fall short of 6 G THz communication requirements on kilometer-level coverage, which demand multiwatt continuous-wave power and a gain greater than 30 dB for practical deployment. Overcoming these limitations necessitates the adoption of innovative approaches, such as advanced beam–wave synchronization techniques with novel SWS design and reduced electron scattering with optimized electrostatic focusing schemes.

Building upon our team’s prior progress in staggered double vane (SDV) SWS, which achieved 1.6 W output power at 335 GHz^26^, a transformative redesign is now realized using novel modified circular bend (MCB) FWG (Fig. 2b). Inspired by the field-confinement mechanism of reentrant cavities^29^, we introduce geometric loading in the curved sections of the FWG SWS to enlarge the waveguide’s bend curvature, which can redistribute the electric field in critical regions. To be specific, by strategically expanding the radius of the outer waveguide arc, the intensity of the axial electric field in the straight region is substantially enhanced. This enhancement directly improves the beam-wave coupling dynamics, thereby maximizing energy-transfer efficiency between the electron beam and electromagnetic waves. This geometric innovation enables advanced coupling impedance values. Simulations (Fig. 2d) confirm that the MCB-based structure achieves 1.7 Ω at 335 GHz, a sixfold leap over the 0.28 Ω performance of our team’s original SDV design. This substantial improvement in coupling impedance experimentally confirms the effectiveness of the reentrant-cavity-inspired field optimization strategy in previous millimeter-wave TWTAs^30,31^. More importantly, the MCB architecture demonstrates potential as a scalable solution for high-power THz amplifiers.

The enhancement of coupling impedance critically determines the achievable gain in TWTAs. The gain parameter formula for TWTAs is given as follows^32^:

1 $$G={\left(\frac{K{I}_{0}}{4{V}_{0}}\right)}^{\frac{1}{3}}$$

where *K* denotes the interaction impedance, *I*_0_ represents the DC beam current, and *V*_0_ stands for the DC beam voltage. It can be derived that higher interaction impedance leads to increased gain parameter *G*. In TWTAs, *G* serves as a fundamental figure of merit that directly influences the gain. Another method to increase output power is to optimize the operating region of the device. Figure 2e shows the Brillouin zone (which is also often referred to as the dispersion curve) diagram of the MCBs with a beam line of 19 kV. The electric field distributions of both the first and second eigenmodes (termed as the first and second modes in Fig. 2e) correspond to the TE_10_ mode. This occurs because the CST E-eigenmode solver, under periodic boundary conditions, samples the dispersion relation at discrete wave numbers (k-vectors), effectively splitting the continuous dispersion curve of the single physical TE_10_ mode into distinct numerical solutions. The vicinity of the intersection point between the 19 kV beam line and the first mode line constitutes the region of electron beam and electromagnetic wave interaction, directly manifesting the operational bandwidth of the TWTA. Usually, the center frequency to cutoff frequency ratio (*f*_0_/*f*_*c*_) is set near 1.25 to balance power and bandwidth^33^. In this study, we optimize the operating point by setting *f*_0_/*f*_*c*_ near 1.10 (*f*_0_ ≈ 339 GHz, *f*_*c*_ ≈ 311 GHz), which moves the working point closer to the 2π point. This strategic adjustment significantly enhances interaction impedance characteristics, thereby substantially improving the output power performance.

The remaining cold characteristics of MCBs are also evaluated, as shown in Fig. 2f and g. The calculated transmission loss of the SWS is about 610 dB m^−1^ at 335 GHz, which is closely related to surface roughness after processing. The value of the normalized phase velocity at 335 GHz is about 0.265, corresponding to a 19 kV operating voltage. The numerical simulations are further conducted using CST Particle Studio, and a solid model is shown in Fig. 2c. A two-section MCB is adopted, with the sections separated by a sever to avoid oscillations. In the first section, SWS modulates the electron beam to initiate electron bunching. As the progressive enhancement of bunching leads to the formation of a deceleration field region, the kinetic energy extracted from the decelerated electron bunches is transferred to the electromagnetic waves, thereby amplifying the electromagnetic wave signal. Figure 2h presents the saturated output power and gain of a 335 GHz TWTA obtained from a beam–wave interaction simulation code. The particle-in-cell results predict an output power of the circuit over 2 W with a bandwidth of 6 GHz, and the maximum gain and saturation output power correspond to 52.8 dB at 334 GHz and 4.76 W at 335 GHz, respectively. In addition, Fig. 2i shows that the corresponding frequency spectrum at 335 GHz has a relatively good purity, with a signal gain of 47.1 dB.

The electron optical system of our continuous-wave TWTA module integrates an optimized Pierce electron gun with a matched PPM focusing system to achieve efficient electron beam bunching. Full simulation methodologies are detailed in Supplementary Information S1.

### Key parameter characterization of the fabricated TWTA

**Fig. 3:**
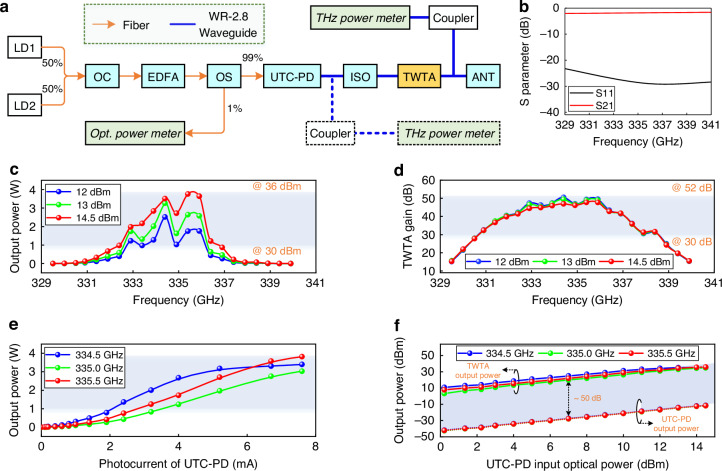
Performance characterization of continuous-wave TWTA. **a** Experimental setup for the test bed. Using heterodyne photomixing, two lightwaves from two tunable lasers generate THz waves in the range of 329–340 GHz through the UTC-PD. An optical power meter is used to measure the UTC-PD input optical power, while both of the THz powers after UTC-PD and TWTA are measured using a THz power meter with a WR-2.8 waveguide interface. **b** S-parameters of the ultrathin diamond RF window measured using a vector network analyzer, where the black curve represents *S*_11_ (reflection characteristics) and the red curve shows *S*_21_ (transmission characteristics). **c** TWTA output power versus the operation THz carrier frequency. The red, green, and blue lines represent different UTC-PD input optical powers (12, 13, and 14.5 dBm, respectively). **d** TWTA gain versus the THz carrier frequency under different UTC-PD input optical powers. **e** TWTA output power versus the operation photocurrent of UTC-PD under three different THz carrier frequencies. **f** Output powers of UTC-PD and TWTA versus the UTC-PD input optical power. EDFA erbium-doped fiber amplifier, OS optical splitter, UTC-PD uni-traveling carrier photodiode, ISO Isolator, ANT antenna

Using the simulation-optimized design parameters of the SWS, high-precision micromilling technology is used to fabricate the core component. For signal transmission, an ultrathin diamond vacuum window structure is adopted, coupled with a multistage depressed collector for energy recovery. Through systematic integration, a high-power continuous-wave TWTA module operating at 335 GHz is successfully developed. The energy window is first tested using a vector network analyzer, and the measured S-parameters are shown in Fig. 3b. The diamond energy window exhibits an *S*_21_ (red curve, representing transmission characteristics) of less than 1 dB over a 10 GHz frequency range (329–341 GHz), demonstrating the low-loss characteristics of the ultrathin diamond window, which is conducive to achieving high-power output in TWTA. *S*_11_ (black curve, representing reflection characteristics) remains below −23 dB, indicating that the diamond window has excellent matching properties. Notably, no sharp peaks in *S*_11_ are observed across the operational bandwidth, confirming the absence of signal reflection during transmission through the energy window.

By optimizing the magnetic field distribution of the PPM focusing system, the beam transmission efficiency of the TWTA is progressively increased to 90%. At this point, the static intercepted current of the TWTA is measured as 4.2 mA, indicating stable continuous-wave operation after aging.

To further systematically characterize the fabricated continuous-wave TWTA module, we build a test bed enabled by photonics, as shown in Fig. 3a. Two tunable laser diodes (LDs) are coupled by a 50/50 optical coupler (OC). After amplification by an erbium-doped fiber amplifier (EDFA), the combined optical lightwaves are divided into two branches using a 1/99 optical splitter (OS). Among them, 1% of the optical power is sent to the optical power meter for real-time optical power monitoring, whereas the remaining 99% is sent to the UTC-PD for O/T conversion. By adjusting the operation carrier frequencies of the two LDs, the corresponding THz wave can be generated within the range of 329–340 GHz. Subsequently, the continuous-wave TWTA under an operating beam voltage of 19 kV and beam current of 28.4 mA is used to amplify the THz wave. To prevent the reflected power from damaging the UTC-PD, we insert a THz isolator (ISO) between the UTC-PD and TWTA. The THz power outputs by both the UTC-PD and TWTA are measured using calorimetric WR-2.8 waveguide average power meters through a 20 dB directional coupler, where just 1% of the energy is output from the coupled port for real-time THz power monitoring.

First, the TWTA output power versus the operation THz carrier frequency curves are given under three kinds of UTC-PD input optical powers: 12, 13, and 14.5 dBm (Fig. 3c). To protect the UTC-PD from damage, its input optical power is controlled to below 14.5 dBm. It can be observed that the TWTA output power can exceed 1 W (i.e., 30 dBm) within the frequency range of 332–337 GHz, with the maximum output power approaching 4 W (~36 dBm) at 335.5 GHz. Next, the TWTA gain versus the operation THz carrier frequency curves are plotted, as shown in Fig. 3d. This TWTA module demonstrates over 30 dB of gain across the 330.5 to 338.5 GHz range (8 GHz bandwidth), reaching a peak gain of ~52 dB at 334.5 GHz. Subsequently, the relationships between the operation photocurrent of UPC-PD and the output power of TWTA are further established at three frequency points around 335 GHz. Figure 3e shows that as long as the operation photocurrent of UPC-PD exceeds 2 mA, the TWTA can achieve an output power of over 1 W. Its peak output power can reach up to 3.82 W with a photocurrent of 7.6 mA at 335.5 GHz. As power efficiency is one of the important indicators for THz transmission systems^34^, we further evaluate the power efficiency of TWTA. The overall power efficiency of TWTA can be calculated as the ratio of its THz output power to the total power consumed by the DC power supplies that energize the amplification process. The latter contains the heater power, thermal power loss due to ithe ntercepted electron beam, and the recovered power from the depressed collector, which are evaluated as 128.2 W in total for our 335 GHz TWTA. As a result, the overall power efficiency of TWTA is calculated as about 3%. This result represents the highest record for a TWTA at this frequency band based on experimental measurement data. Finally, Fig. 3f shows the gain performance of the TWTA around 335 GHz. The results indicate that the continuous-wave TWTA exhibits a good linear dynamic range and achieves an almost flat 50 dB gain amplification under different UTC-PD input optical powers from 0 to 14.5 dBm. Notably, only a slight power saturation is observed from the TWTA with a UTC-PD input optical power above 12 dBm. This means that once the conversion efficiency of the UTC-PD is improved, the peak output power of the TWTA can still be increased, which may reach the simulated 4.76 W shown in Fig. 2h.

### TWTA-based THz wireless communication with a diversity reception scheme

**Fig. 4:**
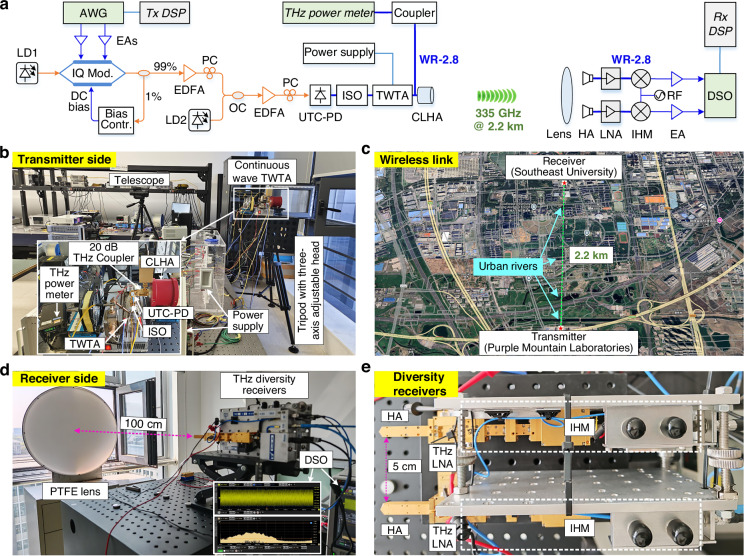
Demonstration of the TWTA-based THz wireless communication system with diversity reception scheme. **a** Schematic of the 335 GHz long-range photonic–electronic converged THz wireless transmission system. The system includes an indoor photonics-assisted THz transmitter and a pair of diversity receivers, as well as an outdoor 2.2 km THz wireless link. **b** Photograph of the high-power photonics-assisted THz transmitter driven by a continuous-wave TWTA. **c** Wireless channel environment of a 2.2 km point-to-point link with several crossing urban rivers. **d** Photograph of the receiver side with a diversity reception scheme. **e** Details of the diversity receivers with two electronics-based THz mixing receivers spaced 5 cm. AWG arbitrary waveform generator, EAs electrical amplifiers, IQ Mod, in-phase (I) and quadrature (Q) modulator, DC bias direct current bias, Bias Contr. bias controller, EDFA erbium-doped fiber amplifier, PC polarization controller, UTC-PD uni-traveling carrier photodiode, ISO isolator, CLHA cylindrical lens horn antenna, HA horn antenna, LNA low-noise amplifier, IHM integrated harmonic mixer, RF radio frequency, DSO digital storage oscilloscope

The self-developed continuous-wave TWTA given above can be used to build high-power THz transmitters, achieving a significant leap in the emission power of photonics-assisted THz communication from the microwatt to the watt level. Other than high-power THz transmitters, high-sensitivity THz receivers are also essential for outdoor long-range THz wireless communication, especially for kilometer-level wireless transmission. Figure 4a shows our experimental setup for a photonic–electronic converged THz long-range wireless communication system, which enables a single-emission and double-reception wireless transmission architecture. It includes an indoor photonics-assisted THz transmitter and a pair of electronics-based THz diversity receivers, as well as an outdoor 2.2 km THz wireless link. The THz transmitter is mainly composed of three components: electro-optic modulation, optical-to-THz conversion, and high-power amplification modules. The complex baseband signal generated by the arbitrary waveform generator (AWG) is fed into an in-phase and quadrature (IQ) modulator for electro-optic modulation, supporting adaptive bias control through an optical feedback loop. Two individual free-running tunable LDs with a frequency interval of 335 GHz serve as an optical carrier (LD1) and an optical local oscillator (LO, LD2), respectively. Then, the modulated optical signal and unmodulated optical LO are combined, amplified, and polarization-aligned before heterodyne photomixing using the UTC-PD, generating a 335 GHz THz signal. The weak THz signal is then boosted by our high-power continuous-wave TWTA with real-time power monitoring (Fig. 4b) and radiated into the air using an integrated cylindrical lens horn antenna (CLHA). Beam alignment between the transmitting and receiving ends is achieved using two tripods with three-axis adjustable heads in conjunction with a telescope. The coarse alignment is realized by flexibly adjusting the position and direction of the tripods at both ends with the assistance of a telescope, while the fine alignment is further performed through the three-axis adjustable head, which is installed on each tripod. An outdoor 2.2 km line-of-sight THz wireless link is established between Purple Mountain Laboratories (Tx) and Southeast University (Rx) in Nanjing, China, as shown in Fig. 4c. There are four urban rivers crossing the wireless link in our field deployment environment.

At the THz wireless receiver side, a circular polytetrafluoroethylene (PTFE) THz lens is deployed to maximize the received THz power by focusing the received THz wave onto the THz antennas behind it (Fig. 4d). To improve the receiving sensitivity, a pair of electronics-based THz mixing receivers spaced 5 cm apart from each other is used behind the PTFE lens. As shown in Fig. 4e, each THz receiver consists of a horn antenna (HA), a THz low-noise amplifier (LNA), and an integrated harmonic mixer (IHM), and both receivers are driven by the same RF source. A real-time digital storage oscilloscope (DSO) is used to capture the downconverted intermediate frequency (IF) signals with a 5 GHz carrier frequency. The further signal recovery is conducted in the offline receiving digital signal processing (DSP) module, where the diversity receiving gain can be achieved through a maximum ratio combining (MRC) operation^35^. The detailed configurations of key device components and the theoretical model of signal-to-noise ratio (SNR) gain for the single-emission and double-reception scheme in this experiment are described in the Methods section. Meanwhile, the detailed link budget for our experimental system is presented in Supplementary Information S2.2.

Notably, our THz system operates in the far-field mode. In addition, although the transceivers are located indoors, the windows at both ends and the building between the links do not shelter from the Fresnel zone. A detailed analysis is given in Supplementary Information S2.1. The transmission performance of this THz link under different times of the day and humidity conditions is given in Supplementary Information S4.2. We also demonstrate the real-time transmission of 5G new radio and high-definition video using the 2.2 km wireless link at 335 GHz employing a single THz receiver (see details in Supplementary Information S4.3). The stable and uninterrupted real-time live video demonstrates the good robustness of our 2.2 km wireless link.

### Performance of diversity reception processing over a 2.2 km THz wireless link

**Fig. 5:**
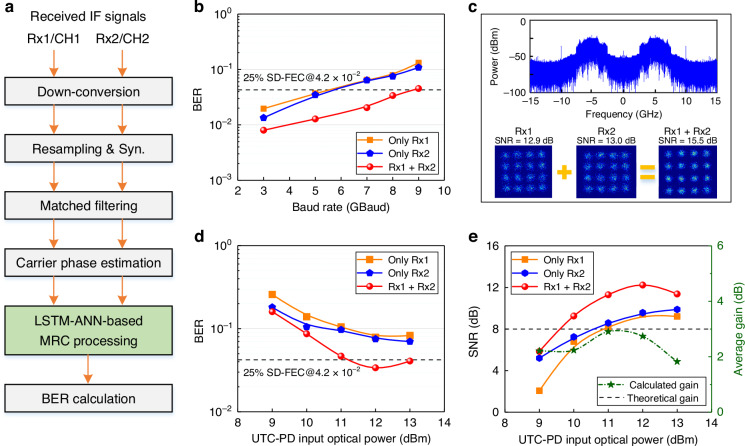
Experimental results of diversity reception processing over a 2.2 km THz wireless link at 335 GHz. All the results below adopt the 16QAM modulation format. **a** DSP flow for signal MRC processing. For the single Rx1 or Rx2 receiving case, one typical third-order Volterra nonlinear equalizer is used instead of merging Rx1 and Rx2 via LSTM–ANN-based MRC processing. **b** BER versus the different symbol rate with a UTC-PD input optical power of 12 dBm. **c** Electrical spectrum of the downconverted IF signal (up figure) and constellation diagrams after signal recovery (down figure) under a fixed symbol rate of 5 GBaud. **d** BER versus the UTC-PD input optical power with a 32 Gbit s^−1^ transmission rate. **e** SNR (left vertical axis) and average gain (right vertical axis) versus the UTC-PD input optical power with a 32 Gbit s^−1^ transmission rate. The theoretical gain for the single-emission and double-reception diversity scheme corresponds to 3 dB (see details in the Methods section), whereas the average gain in our experiment is calculated by subtracting the average SNR of RX1 and RX2 from the SNR obtained after merging Rx1 and Rx2. In this case, the calculated peak gain of SNR reaches up to 2.9 dB

The transmission performance of the diversity reception processing for our photonic–electronic converged THz wireless communication over a 2.2 km link is evaluated. The experimental results are shown in Fig. 5. Three different receiving schemes—Only Rx1, only Rx2, and the proposed diversity reception with two receivers (termed as the Rx1 + Rx2 scheme)—are configured for comparative analysis. Among them, the first two schemes only use a single receiver, whereas the last scheme simultaneously adopts two receivers and merges their signals using the MRC technique^35^. In this experiment, the THz center frequency is set to 335 GHz, and the modulation format is fixed as 16-ary quadrature amplitude modulation (16QAM).

Figure 5a depicts the DSP flow for signal MRC processing at the receiving side. The two 5 GHz IF signals captured by the two channels (CH1 and CH2) of DPO from Rx1 and Rx2, respectively, are first downconverted to the baseband. After resampling, frame synchronization, clock recovery, matched filtering, and carrier phase estimation, the obtained two complex-valued symbol sequences are fed into a long-short-term memory–artificial neural network (LSTM–ANN) equalizer^36^. This LSTM–ANN equalizer, composed of one eight-layer neural network (see details in Supplementary Information S4.1), adopts a data-driven approach to achieve symbol recovery through MRC processing based on time memory and information extraction characteristics. Subsequently, the bit error rate (BER) is calculated according to the output symbol sequences. In addition, for only the Rx1 or Rx2 receiving scheme, except for the adoption of a typical third-order Volterra nonlinear equalizer^37^ to replace the LSTM–ANN equalizer, all other processes are the same as in Fig. 5a.

Using the above-mentioned DSP method, we first evaluate the performance of BER varying with the transmission baud rate at a fixed optical power of 12 dBm launched into the UTC-PD, as shown in Fig. 5b. Because of the limited bandwidth of our TWTA, when the baud rate exceeds 5.5 GBaud, the BER for a single receiver scheme cannot meet the 25% overhead soft-decision forward error correction (SD-FEC) BER threshold (i.e., 4.2 × 10^−2^). However, when the proposed LSTM–ANN-based MRC processing is adopted, the baud rate that meets the 25% SD-FEC BER threshold can be increased to 8.7 GBaud, achieving a 58% improvement in the transmission rate (i.e., from 22 to 34.8 Gbit s^−1^). This mainly benefits from the diversity gain due to the two independent THz receivers^38^. Figure 5c depicts the corresponding electrical spectrum of the downconverted IF signal and constellation diagrams after signal recovery under a fixed symbol rate of 5 GBaud. The constellation diagrams show that the average SNR of the recovered symbols from Rx1 and Rx2 is 12.95 dB, whereas this SNR value reaches 15.5 dB after LSTM–ANN-based MRC processing in the Rx1 + Rx2 case. Therefore, a 2.55 dB gain is presented by the diversity reception scheme. This gain is intuitively reflected by clearer and more focused constellation clusters.

To further quantitatively analyze the advantages of the diversity reception scheme in the 2.2 km THz wireless link, Fig. 5d presents the variation curve of BER with the UTC-PD input optical power at a symbol rate of 8 GBaud (corresponding to a transmission line rate of 32 Gbit s^−1^). A single receiver is simply unable to meet the 25% SD-FEC BER threshold, whereas the diversity reception scheme only requires a UTC-PD input optical power exceeding 11 dBm and even exhibits a dynamic range of ~2 dB. This diversity receiving scheme reduces the best BER from 7.71 × 10^−2^ (average BER of only RX1 and RX2 schemes) to 3.38 × 10^−2^ using the LSTM–ANN equalizer. Figure 5e further shows the SNR (left vertical axis) and average gain (right vertical axis) versus the UTC-PD input optical power with a transmission rate of 32 Gbit s^−1^. The average gain is calculated by subtracting the average SNR of Rx1 and Rx2 from the SNR obtained after merging Rx1 and Rx2. The theoretical gain for the single-emission and double-reception diversity scheme corresponds to 3 dB (see details in Methods). In our experiment, the average gain fluctuates within the range of 1 dB, and the peak gain can reach 2.9 dB, exhibiting a difference of only 0.1 dB from the theoretical gain.

Although our experimental scheme simplifies the diversity reception structure (sharing the main THz link before the receiving THz lens), the proposed techniques are universal for common single-emission and double-reception links with independent end-to-end transmission responses. The issues such as atmospheric turbulence, multipath effects, and partial misalignment are critical for a traditional single-input single-output (SISO) link^39^. In contrast, the THz links using the diversity reception scheme may exhibit better robustness. This is mainly because the impairments are statistically independent for different paths, and the probability of both paths simultaneously undergoing deep fading is significantly lower than that of a SISO link. Moreover, the proposed LSTM-ANN combiner implements performance-driven weighting of the two input paths. This intelligent equalization strategy autonomously optimizes the weighting coefficients to significantly improve the overall transmission performance.

## Discussion

In summary, we have developed a high-power and high-gain TWTA supporting continuous-wave operation at 335 GHz. By reshaping coupling dynamics through the transformative redesign of MCB FWG and optimization of the operating region of MCBs, the output power and gain of continuous-wave TWTA can reach 3.82 W and 52 dB, respectively. This leads to a substantial performance improvement above the 300 GHz band compared with conventional SSPA and previous TWTA components. Furthermore, a simple diversity receiving scheme based on two THz receivers and MRC processing is presented, enabling an improved receiving SNR up to 2.9 dB. Leveraging the advanced continuous-wave TWTA and diversity receiving scheme, we demonstrate an ultra-long-range THz communication over 2.2 km at 335 GHz, supporting a net data rate of up to 27.84 Gbit s^−1^ and a record-breaking rate–distance product of 61,248 Gbit s^−1^ ∙ m for the first time using a photonics-assisted THz generation approach. This work marks a significant step forward for the practical application of high-frequency THz toward kilometer-level wireless transmission, preserving the potential for high-capacity wireless communication and enabling the embedding of THz wireless links into mature optical networks.

Table 1 shows an overview of state-of-the-art high-power and high-gain THz amplifiers beyond 300 GHz. Here, we mainly focus on two typical types of THz amplifiers for communication applications: SSPA and TWTA, while some special high-power components for imaging fields, such as THz wave parametric oscillators operating in pulse mode^40^, are not within the scope of our discussion. The SSPA-based solution above 300 GHz has the potential for tens of gigahertz in terms of bandwidth. However, it faces inherent limitations, typically delivering <50 mW of output power and <30 dB of gain^41–44^. In contrast, the TWTA-based solution exhibits significant advancements toward watt-level THz emission power^25,26,40,45^, presenting great promise in addressing insufficient THz emission power. The China Academy of Engineering Physics 10th Research Institute has demonstrated an instantaneous peak power of 3.1 W near 337 GHz, which was, however, constrained by the design of traditional FWG SWS and electron optical system^25^. A limited bandwidth, together with a 10% duty cycle were achieved, preventing true continuous-wave communication functionality. Although our previous work has achieved a continuous peak output power up to 1.6 W at 344 GHz using an SDV SWS^26^, the attainable output power and amplification gain are ultimately constrained by the low interaction impedance of its slow-wave circuit, preventing further enhancement. Compared with these benchmarks, our 335 GHz TWTA in this paper overcomes key limitations in bandwidth, power, and continuous-wave operation through three coordinated innovations: a redesigned FWG SWS structure that suppresses band-edge oscillations^46^ for wider bandwidth; enhanced axial field strength in the interaction region for higher output power; and a beveled modulating electrode that enables stable electron beam transmission for continuous-wave operation. These synergistic advances collectively achieve breakthroughs in TWTA performance, transforming the TWTA into a practical radiation source for long-range THz communications. Our technological leadership can be established by the following three critical advances: (1) A 3.8 W peak output power above 300 GHz is achieved, representing the current highest level. This paves the way for high-frequency THz long-range wireless transmission toward kilometer levels. (2) The maximum signal gain is enhanced to as high as 52 dB, which is twice the current level. Such a high gain allows for a tight combination of UTC-PD-based photonics-assisted THz generation with TWTA-based high-gain amplification. (3) The above high output power and high amplification gain are realized in a continuous-wave operation mode. Nevertheless, technical challenges persist, including significant gain variations across operational bandwidths and restricted bandwidth coverage. Subsequent research should focus on optimizing power–bandwidth capacity and improving gain consistency to meet stringent communication requirements while matching the large-bandwidth and high-capacity characteristics of photonics-assisted THz communications.

**Table 1:** Overview of high-power and high-gain THz amplifiers beyond 300 GHz

Ref.	Type	Device technology	Frequency band (GHz)	Max power (mW)	Max gain (dB)	Duty cycle
^41^	SSPA	InGaAs mHEMT^a^	280–310	6.76	19	None
^42^	SSPA	InP HBT^b^	290–308	10	23.5	None
^43^	SSPA	InP HEMT^c^	280–328	23.4	26	None
^44^	SSPA	InP HEMT	250–300	49.8	26.6	None
^45^	TWTA	VED^d^	315–325	130	19.6	10%
^25^	TWTA	VED	335–338	3100	26.2	10%
^26^	TWTA	VED	330–355	1600	22	100%
**This work**	**TWTA**	**VED**	**330**–**340**	**3820**	**52**	**100%**

^a^*mHEMT* metamorphic high-electron-mobility transistor, ^b^*HBT* heterojunction bipolar transistor, ^c^*HEMT* high-electron-mobility transistor, ^d^*VED* vacuum electronic device

Table 2 presents representative works on long-range THz wireless transmission beyond 300 GHz using electronics^5,47,48^ and photonics^49–53^ THz generation technical routes. For electronics-based schemes, the generated THz signals through RF multiplier chains have relatively high output power in the 300 GHz band and above, allowing for greater wireless transmission distances than those achieved using photonics-assisted schemes. However, the electronic bottleneck inherently limits the transmission rate and operating THz carrier frequency. In contrast, photonics-assisted schemes can enable high-frequency, large-bandwidth, and ultra-high-speed wireless transmission, but the wireless transmission distance is often limited to within several hundred meters. Moreover, the use of an optical frequency comb to generate a THz signal can also avoid the additional phase noise introduced by the frequency multiplication operation in electronics-based schemes, thus supporting the evolution of THz communication toward higher-order modulation^54–56^. Because there is a compromise between the data rate and wireless transmission distance, the rate–distance product is an impartial indicator for evaluating long-range THz communication systems. In previous studies^5^, electronic-based schemes support 300 GHz THz wireless transmission with a net rate of 44.8 Gbit s^−1^ over 1000 m, achieving a rate–distance product of 44,800 Gbit s^−1^ ∙ m. For photonics-assisted schemes, a rate–distance product comparable to that of the electronic schemes is also achieved at 300 and 320 GHz bands, respectively^52,53^. However, the maximum wireless distance is limited to within 850 m. Benefiting from the high-power continuous-wave TWTA and high-sensitivity diversity receiving scheme, this paper realizes an unprecedented rate–distance product up to 61,248 Gbit s^−1^ ∙ m with a 2.2 km breakthrough in the THz wireless transmission distance at 335 GHz for the first time. The limited operating bandwidth and uneven gain of continuous-wave TWTA are the main factors restricting the transmission rate in our long-range THz wireless link. We can expect the transmission rates to exceed 100 Gbit s^−1^ or more as long as the continuous-wave TWTA improves in bandwidth and gain flatness.

**Table 2:** Overview of long-range THz wireless communication performances beyond 300 GHz

Ref.	Technical route^a^	Frequency (GHz)	Modulation format	Wireless distance (m)	Net rate (Gbit s^−1^)	Rate–distance product (Gbit s^−1^ ∙ m)
^47^	Electronics	300	QPSK	500	16	8000
^48^	Electronics	300	32QAM	645	12.6	8127
^5^	Electronics	300	4QAM	1000	44.8	44,800
^49^	Photonics	335	16QAM	400	25.6	10,240
^50^	Photonics	300	16QAM	110	115	12,650
^51^	Photonics	300	QPSK	200	187.5	37,500
^52^	Photonics	320	16QAM	850	50	42,500
^53^	Photonics	300	32QAM	214	205.6	43,998
**This work**	**Photonics**	**335**	**16QAM**	**2200**	**27.84**	**61,248**

^a^The technical route here mainly depends on the THz generation method. Notably, the photonics THz generation scheme may also use the electronics-based THz receiver at the receiving end

The potential operating frequency bands and routes adopted for kilometer-level THz wireless transmission are issues worthy of attention. In March 2019, the US Federal Communications Commission (FCC) has approved the first regularization of the electro-magnetic spectrum for sub-THz (100–300 GHz) and THz (300-3000 GHz) bands^57^. Over 60 GHz of non-consecutive bandwidth is allocated for unlicensed services at frequencies between 100 and 300 GHz, while a wider bandwidth of around 116 GHz is allocated in the 300–450 GHz band. The consideration of frequency band involves a classic trade-off: lower frequencies favor longer distances due to smaller path loss, while higher frequencies offer access to wider contiguous bandwidths for ultimate capacity, albeit over shorter ranges. For the sub-THz band, the frequencies of around 140 GHz and 220 GHz are two important long-range wireless communication windows. Take the operating frequency of 220 GHz as an example. Some previous works have already achieved representative kilometer-level THz wireless transmission using both electronics and photonics THz technical routes. For instance, a real-time transmission of uncompressed 8 K ultrahigh-definition video with 84 Gbit s^−1^ over 1260 m at the 220 GHz has been successfully developed and demonstrated based on all-solid-state electronic components, presenting a rate–distance product of 105,840 Gbit s^−1^ ∙ m^58^. Additionally, a long-range optical-THz-optical link enabled by photonics has also been demonstrated with a 160 Gbit s^−1^ data rate and a 1400 m wireless distance at the 226 GHz, achieving a rate–distance product as high as 224,000 Gbit s^−1^ ∙ m^59^. Although the photonics approach demonstrates superior performance, the electronics solution still holds undeniable competitiveness at present, considering the maturity and integration level of sub-THz solid-state components. However, as the operating frequency extends to 300 GHz and beyond, the limitations of electronic solutions become prominent. Issues like limited modulation rate due to electronic bottleneck constraints, the aggravated multiplicative noise and conversion loss from multi-stage frequency multiplier chains, and increased integration difficulties increasingly emerge. It is under these challenges that our proposed hybrid photonic–electronic synergy solution presents a viable path for achieving long-range THz communication beyond 300 GHz windows. Further expanding the bandwidth of TWTA to match the advantages of photonics-assisted THz generation approach and achieving more compact optoelectronic integration are the key to advancing the future application of this photonic–electronic converged scheme.

## Materials and methods

### Fabrication and characterization of MCBs of continuous-wave TWTA

The loss of SWS exhibits strong dependence on surface roughness stemming from fabrication processes. Increased surface roughness elongates the high-frequency current path, increasing conductor losses. This effect becomes pronounced when the root-mean-square roughness exceeds the skin depth *δ* = *sqrt*(2/*ωμσ*), where *ω* is the angular frequency, *μ* stands for permeability, and *σ* represents conductivity. Consequently, the SWS performance imposes stringent machining precision requirements. To reduce surface roughness, the cold-test component of MCBs is fabricated using an ultraprecision five-axis computer numerical control milling technology. This fabrication system achieves micron-scale positional accuracy and repeatability, enabling the micromachining of fine microstructures and complex three-dimensional features through mechanical material removal, a capability surpassing conventional mechanical machining processes. The surface roughness test results are shown in Supplementary Information S1.3. Achieving a surface roughness of Ra 0.05 μm substantially suppresses conductor losses by minimizing current path perturbation.

### Theoretical model of SNR gain for a single-emission and double-reception diversity scheme

Suppose *s*_0_(*t*) = *s*(*t*) + *n*_0_(*t*) represents the received signal after the PPTE THz lens, where *s*(*t*) and *n*_0_(*t*) are information-bearing and noise components. *n*_1_(*t*) and *n*_2_(*t*) represent the corresponding additive white Gaussian noise introduced by two independent THz receiving channels, respectively. Thus, the received signal for channel 1 or 2 can be given as *s*_*i*_(*t*) = *s*(*t*) + *n*_0_(*t*) + *n*_*i*_(*t*), where *i* = 1, 2. Note that the mean values of the signal and noise components are all zero, and the three noise components are uncorrelated with each other; that is, *E*[*n*_0_*n*_1_] = *E*[*n*_0_*n*_2_] = *E*[*n*_1_*n*_2_] = 0, where *E*[*x*] stands for the expectation of *x*. We merge the two receiving signals with a weight factor *w* (*w*∈ℝ^+^). Then, the resultant signal can be given as *s*_*c*_(*t*) = *s*_1_(*t*) + *ws*_2_(*t*) = (1 + *w*)*s*(*t*) + *n*_0_(*t*) + *n*_1_(*t*) + *w*[*n*_0_(*t*) + *n*_2_(*t*)]. Among them, the signal power is *P*_*s*_ = (1 + *w*)^2^*E*[*s*^2^], and the noise power corresponds to *P*_*n*_ = *E*[(*n*_0_ + *n*_1_ + *w*(*n*_0_ + *n*_2_))^2^] = (1 + *w*)^2^*E*[*n*_0_^2^] + *E*[*n*_1_^2^] + *w*^2^*E*[*n*_2_^2^]. The SNRs after the PPTE lens, channel 1 and 2 reception, and merging processing are described as *SNR*_0_, *SNR*_1_, *SNR*_2_, and *SNR*_*c*_, respectively. *SNR*_*c*_ = *P*_*s*_/*P*_*n*_ can be expressed by *SNR*_0_, *SNR*_1_, and *SNR*_2_ through the following three steps.

### Step 1

Using SNR to represent the noise power. According to the definition of SNR, the relationship between noise power and SNR can be expressed as *E*[*n*_0_^2^] = *E*[*s*^2^]*SNR*_0_^−1^, *E*[*n*_1_^2^] = *E*[*s*^2^](*SNR*_1_^−1^ − *SNR*_0_^−1^) and *E*[*n*_2_^2^] = *E*[*s*^2^](*SNR*_2_^−1^ − *SNR*_0_^−1^).

### Step 2

Solving the optimal weight coefficient *w* using the SNR optimization problem. As *SNR*_*c*_(*w*) = *P*_*s*_/*P*_*n*_ = (1 + *w*)^2^/[2*wSNR*_0_^−1^ + *SNR*_1_^−1^ + *w*^2^*SNR*_2_^−1^], with the derivative being zero under extreme value conditions (i.e., *SNR*_*c*_^−1^(*w*) = 0), we can calculate the optimal weight coefficient *w* = *E*[*n*_1_^2^]/*E*[*n*_2_^2^] = (*SNR*_1_^−1^ − *SNR*_0_^−1^)/(*SNR*_2_^−1^ − *SNR*_0_^−1^).

### Step 3

Substituting the expression of *w* into *SNR*_*c*_ and then simplifying it. According to the detailed derivation in Supplementary Information S3, *SNR*_*c*_ can be given as

2 $$SN{R}_{c}=\frac{SN{R}_{0}^{2}(SN{R}_{1}+SN{R}_{2})-2SN{R}_{0}SN{R}_{1}SN{R}_{2}}{SN{R}_{0}^{2}-SN{R}_{1}SN{R}_{2}}$$

Because of the large propagation loss after 2.2 km wireless transmission, the power of the received THz signal is quite weak. Thus, the receiving noise introduced by channel 1 or 2 dominates the system performance, leading to an obvious reduction in SNR. In this case, *SNR*_0_ >>*SNR*_*i*_ (*i* = 1, 2). We define the SNR gain as the ratio between the merging *SNR*_*c*_ and the mean value of *SNR*_1_ and *SNR*_2_, i.e., Δ*SNR* = *SNR*_*c*_/mean(*SNR*_1_, *SNR*_2_). The maximum achievable SNR gain can be given as

3 $$\Delta SN{R}_{c}{|}_{\max }=\frac{\mathop{\lim}\limits_{SN{R}_{0}\to \infty }SN{R}_{c}}{{\mathrm{mean}}(SN{R}_{1},SN{R}_{2})}=\frac{SN{R}_{1}+SN{R}_{2}}{(SN{R}_{1}+SN{R}_{2})/2}=2$$

Therefore, this single-emission and double-reception scheme can achieve a theoretical SNR gain up to 3 dB, which agrees well with the conclusion of a previous study^60^.

### Detailed configuration of key device components in this experiment

Our experimental setup is shown in Fig. 4a. Two independent external cavity lasers with a linewidth less than 100 kHz serve as the optical carrier (1549.315 nm) and optical LO (1551.995 nm). An 8 Gbaud 16QAM signal with a roll-off of 0.01 is modulated on the optical carrier, and one J-band (260–400 GHz) UTC-PD (NEL, IODPMJ-13001) is employed in optical-to-THz conversion. The UTC-PD has a typical responsivity of 0.2 A W^−1^ and an output power of −11.7 dBm at 335 GHz. After being amplified by the high-power TWTA module, the emission power of the 16QAM THz signal can reach 34.8 dBm. This THz signal is radiated into the air via an integrated CLHA (Anteral, LHA-HG-WR2.8) with a gain of 48.5 dBi and a 3 dB beamwidth of 0.7°. After 2.2 km THz wireless transmission, the estimated beamwidth at the receiving end is close to 26.9 m (2 × tan0.35° × 2200 m). To minimize energy loss as much as possible, based on the available components in the laboratory, one PTFE lens with a diameter of 60 cm and a focus length of 100 cm is used to collect as much THz power as possible and focus it onto the receiving THz HAs at the receiving side. This PTFE lens, together with the receiving antenna, can provide a total gain of 58 dBi. In addition, two solid-state THz LNAs (RPG, H-LNA 250-350) with a gain of 22 dB are employed to amplify the THz signals received from channels 1 and 2, respectively. Then, each THz signal is downconverted into a 5 GHz IF signal using one IHM (VDI, MixAMC 367) THz receiver with a ×9 frequency multiplier chain. Finally, the obtained IF signal in each branch is amplified by an electrical amplifier with a gain of 35 dB before being captured by the real-time DSO.

## Supplementary information

Supplementary Information for Surpassing kilometer-scale terahertz wireless communication beyond 300 GHz enabled by hybrid photonic–electronic synergy

## Data Availability

All data are available in the main text or the Supplementary Information.
