# Supplementary material for: Surpassing kilometer-scale terahertz wireless communication beyond 300 GHz enabled by hybrid photonic–electronic synergy
Source: Light Sci Appl. 2026 May 9;15:228. doi: 10.1038/s41377-026-02321-6 (PMC13157508; doi:10.1038/s41377-026-02321-6)
Supplement: Supplementary file 1 — Supplementary Information for Surpassing kilometer-scale terahertz wireless communication beyond 300 GHz enabled by hybrid photonic–electronic synergy [file 41377_2026_2321_MOESM1_ESM.docx]

**Supplementary Information for**

**Surpassing kilometer-scale terahertz wireless communication beyond 300 GHz enabled by** **hybrid photonic–electronic synergy**

Yuancheng Cai^1,2^†, Lin Zhang^3^†, Jiao Zhang^1,2^†, Bingchang Hua^1^†, Kexin Ma^3^, Junjie Ding^1^, Xingwang Bian^3^, Mingzheng Lei^1^, Yingzhou Liu^3^, Jiankang Li^2^, Zhigang Xin^1,2^, Xingyu Chen^1^, Jun Cai^3^, Pan Pan^3^, Yongming Huang^1,2^, Jinjun Feng^3^*, Min Zhu^1,2^* and Xiaohu You^1,2^*

*^1^ Purple Mountain Laboratories, Nanjing 211111, China.*

*^2^ National Mobile Communications Research Laboratory, Southeast University, Nanjing 210096, China.*

*^3^ National Key Laboratory of Science and Technology on Vacuum Electronics, Beijing Vacuum Electronics Research Institute, Beijing 100015, China.*

* E-mail: fengjinjun@cetc.com.cn; [minzhu@seu.edu.cn](mailto:minzhu@seu.edu.cn); [xhyu@seu.edu.cn](mailto:xhyu@seu.edu.cn)

† These authors contributed equally to this work.

**This Supplementary Information consists of the following sections:**

S1. Design of a 335 GHz continuous-wave TWTA

S1.1. Thermal velocity effects in 335 GHz electron guns

S1.2. Design of an electron optical system for 335 GHz TWTA

S1.3. Supplementary machining and testing of the SWS cold-test component

S1.4. Phase noise measurement for 335 GHz TWTA

S2. Power budget for a 2.2 km long-range THz wireless link

S2.1. Field region analysis

S2.2. THz power budget

S3. Theoretical model for a single-emission and double-reception diversity scheme

S4. Supplementary information for long-range THz wireless communication

S4.1. Detailed flowchart for the LSTM–ANN equalizer

S4.2. Performance under different times of the day and humidity conditions

S4.3. Real-time demonstration of 5G NR and live video transmission

**S1.** **Design of a 335 GHz continuous-wave traveling wave tube amplifier (TWTA)**

**S1.1. Thermal velocity effects in** **335 GHz electron guns**

Electron beam formation and transport are critical for designing a high-power continuous-wave TWTA. Although TWTAs offer superior power and gain advantages even at terahertz (THz) bands^1,2^, achieving multiwatt operation necessitates electron beam current densities exceeding 4.7 A cm^−2^ with sub-0.1 mm beam waists, a regime where thermal velocity electron emission effects dominate beam dynamics^3,4^. These effects are exacerbated in miniaturized electrostatic focusing systems required for wideband THz operation, particularly when using high-compression electron guns with microscale emitter diameters.

The designed Pierce-type electron gun features an M-type cathode, tunable focus electrode, and ion-suppression anode (+200 V bias). It generates a 19 kV, 30 mA pencil beam with an 85 μm waist radius, and a 0.76 fill factor. As demonstrated in Fig. S1a, thermal velocity distributions fundamentally alter beam current profiles. Idealized simulations neglecting thermal effects predict dual-peaked current densities—peaking at 3400 A cm^−2^ (point A) and 5300 A cm^−2^ (point B). In contrast, thermal-inclusive models exhibit single-peaked distributions with 92% lower maximum density (point C: 420 A cm^−2^). This degradation stems from the differential convergence of emitter subregions. The outermost electrons undergo initial bunching under electrostatic field confinement. Without transverse expansion induced by thermal velocity spread, they subsequently form a current density peak at location A and then a second current density peak at point B, with inner and middle layer electrons (Fig. S1b). There is only one peak at point C because three-layer electrons experience stronger electrostatic focusing but suffer from greater thermal dispersion, considering thermal velocity effects.

Thermal velocities further degrade transverse beam confinement. Comparative analyses of transverse velocity distributions (Figs. S1c and S1d) show that thermal effects broaden velocity spreads by nearly an order of magnitude, increasing maximum transverse velocities by 25% (Fig. S1e). Concurrently, axial velocity distributions exhibit enhanced scattering, reducing phase-space coherence critical for efficient beam–wave interaction.

The above results underscore the fundamental trade-off between the beam compression ratio and thermal-velocity-induced emittance growth in electrostatic-focused THz electron guns. Mitigating such effects requires advanced emitter designs and dynamic focusing schemes to achieve sub-0.1 mm beam waists while maintaining >95% transmission efficiency, which are prerequisites for practical high-power THz TWTAs.


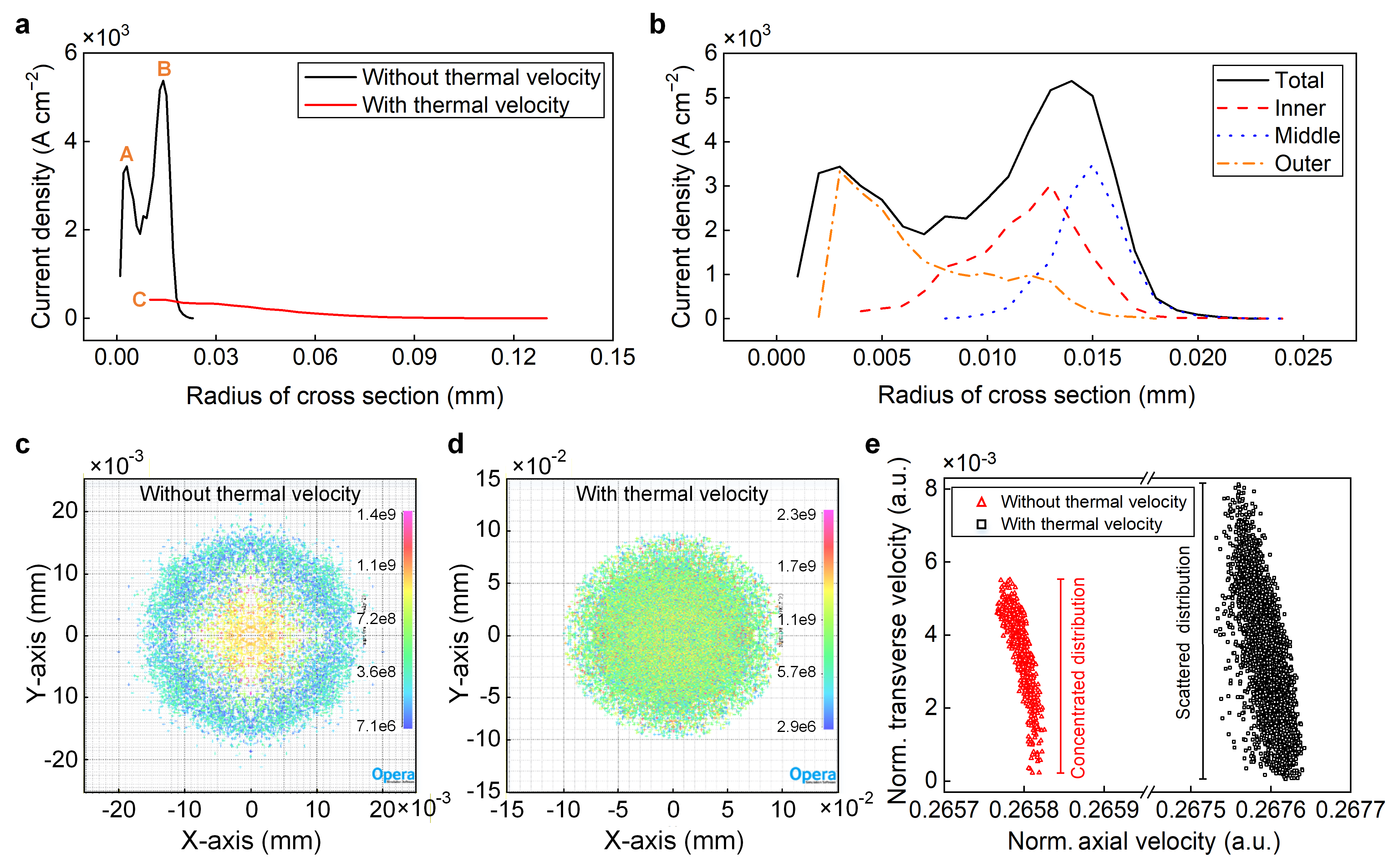


**Fig. S1 | Effects of thermal velocity in 335 GHz electron guns.** **a,** Distribution of current density at various radial positions. **b,** Distribution of current density at different layers. The surface of the cathode is divided into three layers of equal area—an inner layer, a middle layer, and an outer layer—to observe the uniformity of electron emission. **c,** Transverse velocity distributions at the beam waist without the influence of thermal initial velocity. **d,** Transverse velocity distributions at the beam waist with the influence of thermal initial velocity. **e,** Comparison of the normalized transverse velocity and axial velocity values with the speed of light. The normalization operation is achieved by dividing the actual velocity by the speed of light in vacuum.

**S1.2. Design of an electron optical system for 335 GHz TWTA**

To overcome the influence of thermal initial velocity, two primary design approaches are implemented.

The first technical approach involves reducing the electron beam compression ratio to suppress the increase in transverse velocity caused by excessive beam compression^3^. The electron beam compression ratio quantifies the radial convergence of an electron beam from the cathode emission surface to the minimum beam waist. Mitigating thermal velocity spread effects is primarily achieved through three approaches: reducing the cathode emission temperature, elevating the anode voltage, and decreasing the beam compression ratio^3^. Among these, the reduction of the beam compression ratio is the most effective method for suppressing transverse beam expansion. By diminishing the cathode diameter from 0.55 to 0.40 mm while maintaining the beam waist radius at ~0.08 mm, the compression ratio is reduced from 47 to 25—representing a nearly twofold decrease. Following compression ratio reduction, the electron transmission efficiency increases from 95% to 99.7%.

The second method involves optimizing the focusing electrode structure. In conventional Pierce-type electron guns, the focusing electrode forms a tilt angle of 67.5° with the cathode emission plane^5^. In this work, the inclination angle between the focusing electrode and the cathode plane is optimized to 47°, enabling improved electrostatic field uniformity and mitigating premature overcompression of out-layer electrons. An assembly drawing of the electron gun is shown in Fig. S2a.

The electron beam focused by the magnetic field is shown in Fig. S2b, incorporating both aforementioned optimizations. Equipotential line analysis reveals that the combined electrostatic fields of the focus electrode and anode generate a converging lens structure, resulting in significant electron beam compression. This configuration elevates the beam current density from 4.7 to 132 A cm^−2^. Subsequently, the beam enters the electron beam tunnel of the slow-wave structure (SWS) at an axial position of 14 mm, where a confining axial magnetic field (6,000 Gs) enforces a uniformly oscillating, long-range stable beam envelope. Consequently, over 99.7% of electrons actively engage in energy exchange with the electromagnetic wave, enabling continuous-wave operation of the TWTA through sustained beam–wave synchronization.


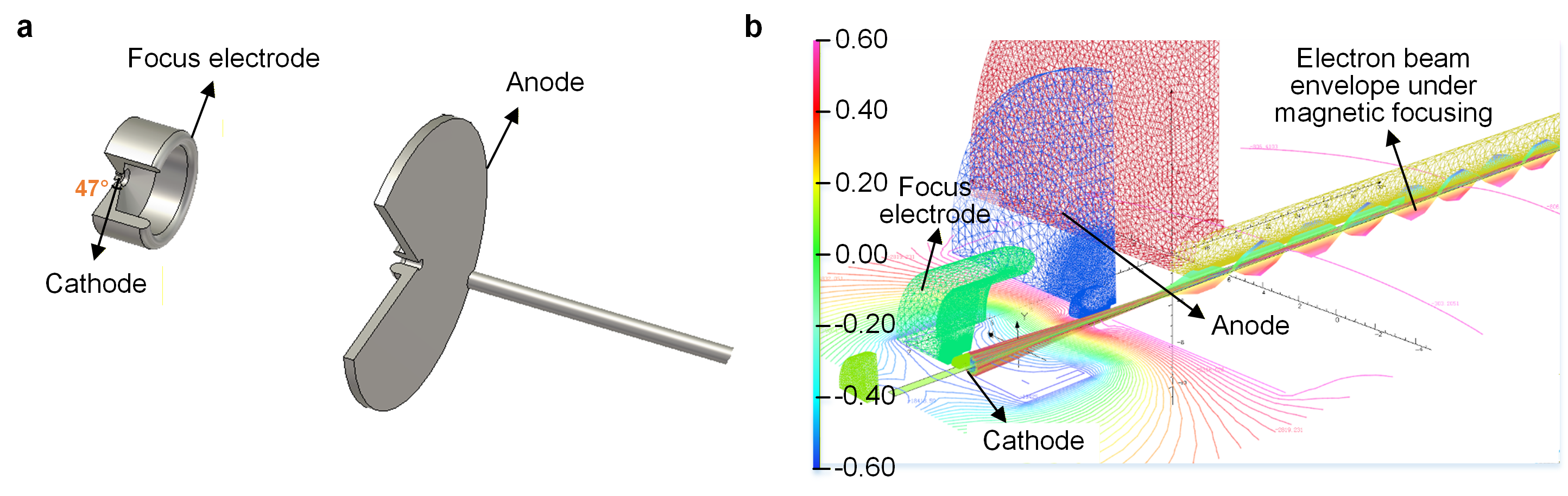


**Fig. S2 | Electron gun and beam bunching under the electron optical system. a,** Assembly drawing of the electron gun, with an inclined plane focusing electrode. **b,** Electron beam focused by the magnetic field, achieving a 99.7% beam transmission ratio.

**S1.3. Supplementary machining and testing of the SWS cold-test component**


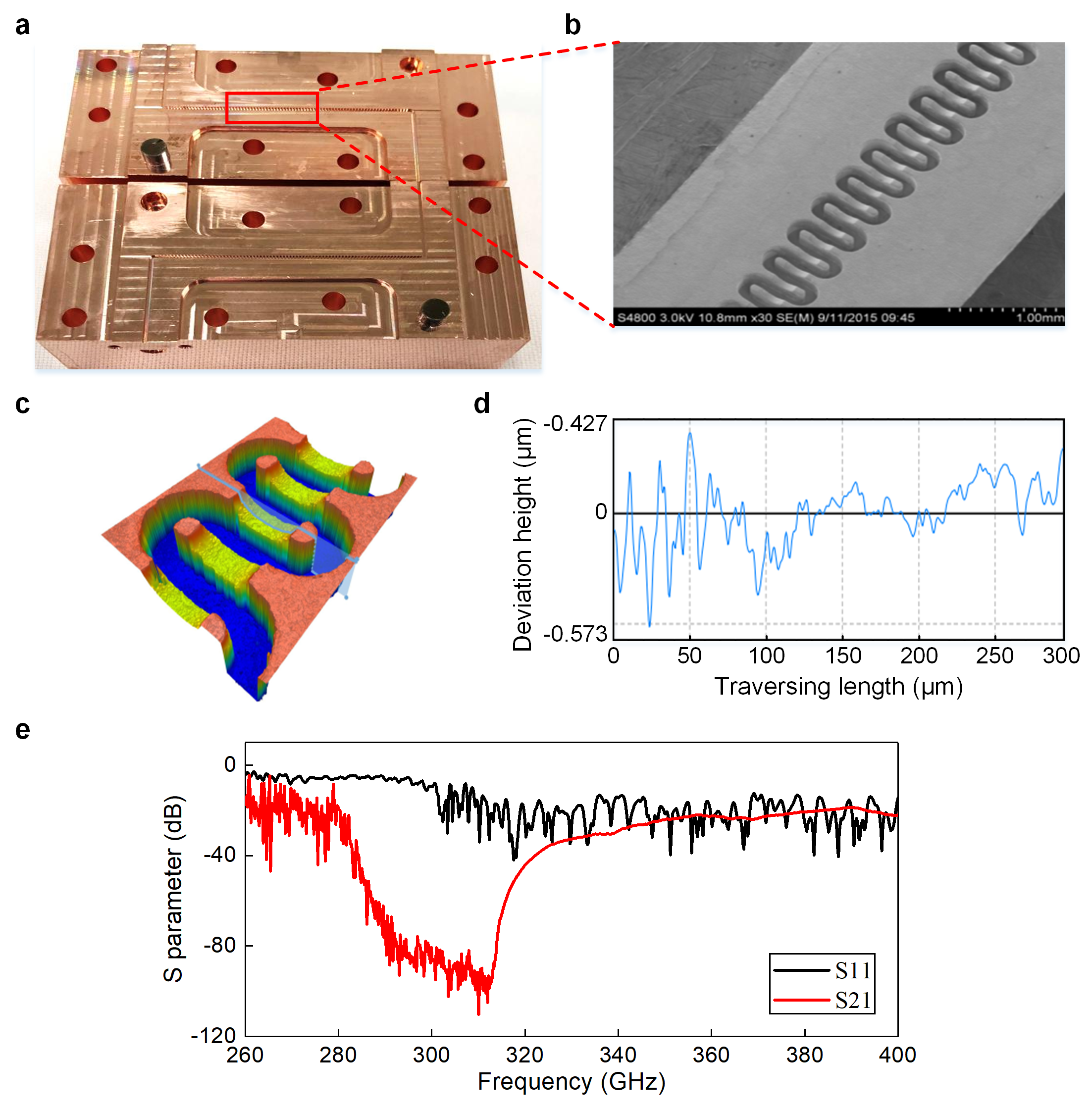


**Fig. S3 | Physical image of the cold-test component, microscopic structure under scanning electron microscope, surface roughness, and S-parameter measurement results.** **a,** Cold-test component processed on a copper substrate with a magnified view of local circuit microstructure. **b,** Microscopic schematic of the slow-wave circuit and **c,** corresponding surface roughness. **d,** Height deviation profile acquired via stylus profilometry over a specified traverse length across the SWS surface. **e,** S-parameter measurement results of the cold-test component, where the black curve represents S_11_ and the red curve denotes S_21_.

To characterize the machining accuracy of the SWS, a cold-test component is fabricated using micromilling techniques, as shown in Fig. S3a. Fig. S3b presents a scanning electron micrograph of the fabricated SWS, exhibiting a groove width of 100 μm with an aspect ratio as high as 3:1. A dimensional tolerance of ≤5 μm and a sidewall verticality of >89.2° are achieved using precision high-speed micromilling. High-precision machining ensures dimensional conformity to design tolerances in the SWS. This dimensional consistency guarantees a uniform phase velocity of electromagnetic wave propagation along the SWS, enabling stable beam–wave energy transfer with the electron beam. Scanning electron microscope characterization reveals a surface roughness below 50 nm, as shown in Fig. S3c. Fig. S3d displays the height deviation profile acquired via stylus profilometry over a specified traverse length across the SWS surface, quantitatively characterizing the surface roughness of the SWS. This submicron surface quality effectively reduces ohmic losses in the SWS, thereby significantly enhancing the output power performance of the 335 GHz TWTA. The S-parameter measurements of the cold-test component demonstrate an insertion loss of ~20 dB. Given the 33 mm physical length of the test structure, the calculated loss of the slow-wave circuit reaches 606 dB m^−1^, showing good agreement with the simulation results shown in Fig. 2f in the main manuscript.

**S1.4.** **Phase noise measurement** **for 335 GHz TWTA**

To measure the phase noise performance of our 335 GHz TWTA, a test bed based on the electronic up and down frequency conversion scheme as shown in Fig. S4a is built for simplicity. A 13.96 GHz radio frequency (RF) signal is first produced by a 9 kHz – 20 GHz signal generator, and then is converted to around 335 GHz THz signal using a frequency multiplier (FM) with a multiplication factor of 24. This THz signal is fed into the TWTA via its input window, and its output THz signal is passed through a 20 dB directional coupler before being delivered to the extension module (EM) of spectrum analyzer. The extension module converts the 335 GHz THz signal to a 425 MHz intermediate frequency (IF) signal, which is then available to be measured the phase noise using a spectrum analyzer. The photograph of phase noise measurement experimental setup is illustrated in Fig. S4b.

We first test the phase noise of the received IF signal without going through TWTA and a 20 dB coupler, whose result actually represents the phase noise of the test link, mainly including the intrinsic noise carried by original RF signal and the frequency multiplication noise introduced by frequency multiplier chains embedded in FM and EM units. It can be seen from Fig. S4c that the noise floor of test link (i.e., only FM + EM case) is close to –115 dBc Hz^−1^. After introducing the 335 GHz TWTA, retest the noise of the received IF signal and compare it with the previous test results. We find that the phase noise curves of the two basically coincide within an offset frequency of 100 kHz. This means that the system phase noise in this region is primarily attributed to the test link. However, as the offset frequency continues to increase, the impact of phase noise introduced by TWTA gradually becomes prominent. The corresponding noise floor after using TWTA (i.e., FM + TWTA + EM case) is below –80 dBc Hz^−1^, which raises the noise floor by over 30 dB as compared with the link without using TWTA. This is mainly due to the ripple of the high-voltage power supply and shot noise of the electron beam of TWTA.


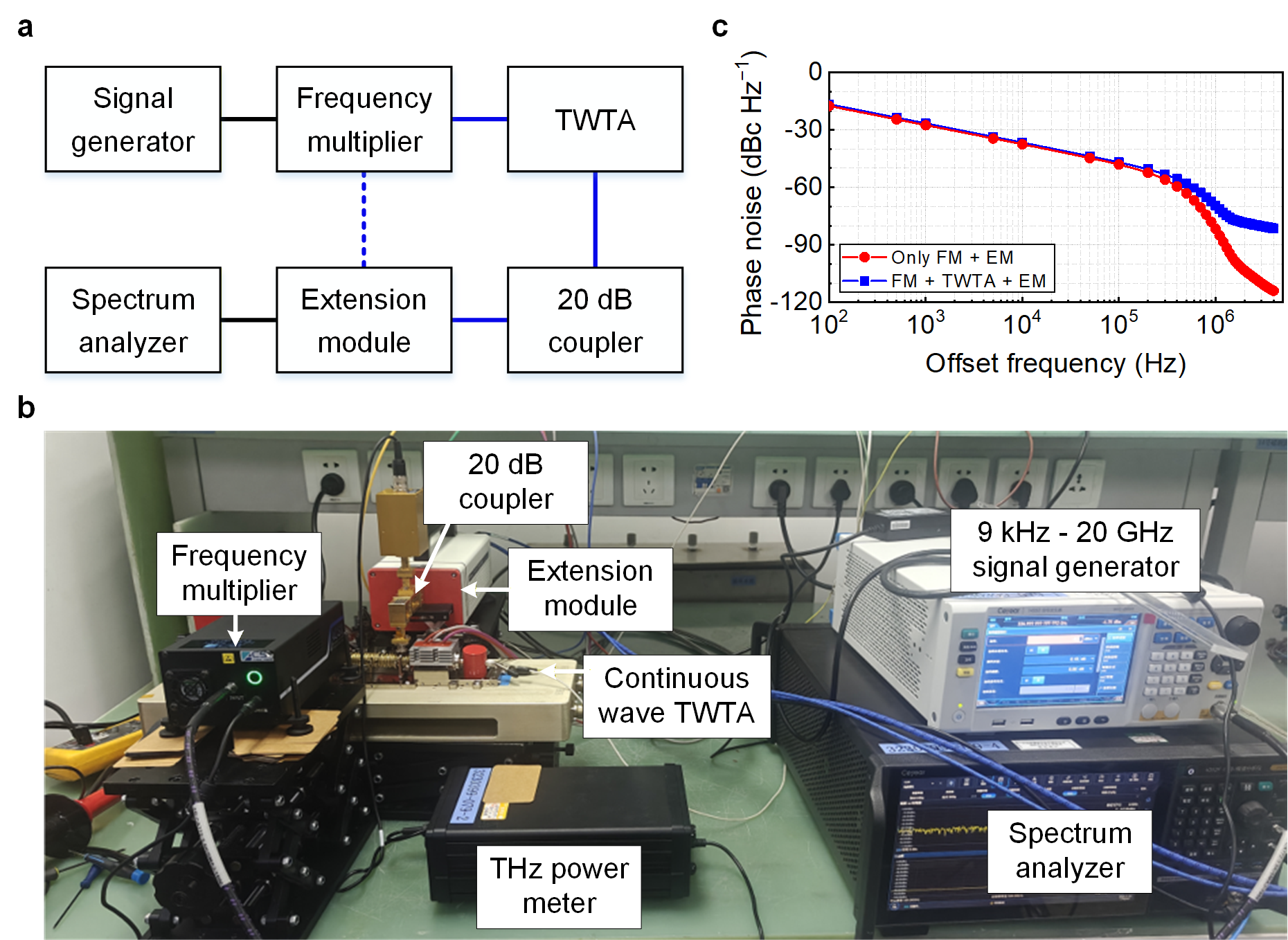


**Fig. S4 | Phase noise** **measurement** **for 335 GHz TWTA. a,** Experimental setup for the test bed. **b,** Photograph of experimental setup. **c,** Phase noise measurement results. FM, frequency multiplier; EM, extension module.

**S2. Power budget for a 2.2 km long-range THz wireless link**

**S2.1.** **Field region analysis**

In this section, we first analyze the operation field region of the THz communication system before studying the power budget of the THz link. The Fresnel and Fraunhofer boundaries demarcate the operational field regions of an antenna into the reactive near-field, radiating near-field, and far-field regions, progressing from near to far. According to the scalar diffraction theory, the Fraunhofer boundaries can be expressed as follows^6^:

$d_{Fraunhofer}=\frac{2D^{2}}{\lambda}$ (S1)

where *D* is the maximum aperture dimension of the antenna and *λ* is the wavelength of the THz wave.

Fig. S5a shows the schematic of the 2.2 km THz wireless link between the integrated cylindrical lens horn antenna (CLHA) at the transmitter and the THz polytetrafluoroethylene (PTFE) lens at the receiver. The transmitter and receiver (see Figs. S5b and S5c, respectively) are placed indoors in two different buildings, approximately horizontal with a negligible inclination angle of <0.5°. At the transmitter, the THz signal amplified by the continuous-wave TWTA is converted into a wireless electromagnetic wave via CLHA and then enters the outdoors through the first window, which has a size of 128 cm × 46 cm. At the receiver, the arriving THz electromagnetic wave enters the room through a second window with a size of 100 cm × 70 cm. The distance between the CLHA/PTFE lens and the window is 70/30 cm, whereas the distance between the two windows is ~2200 m.


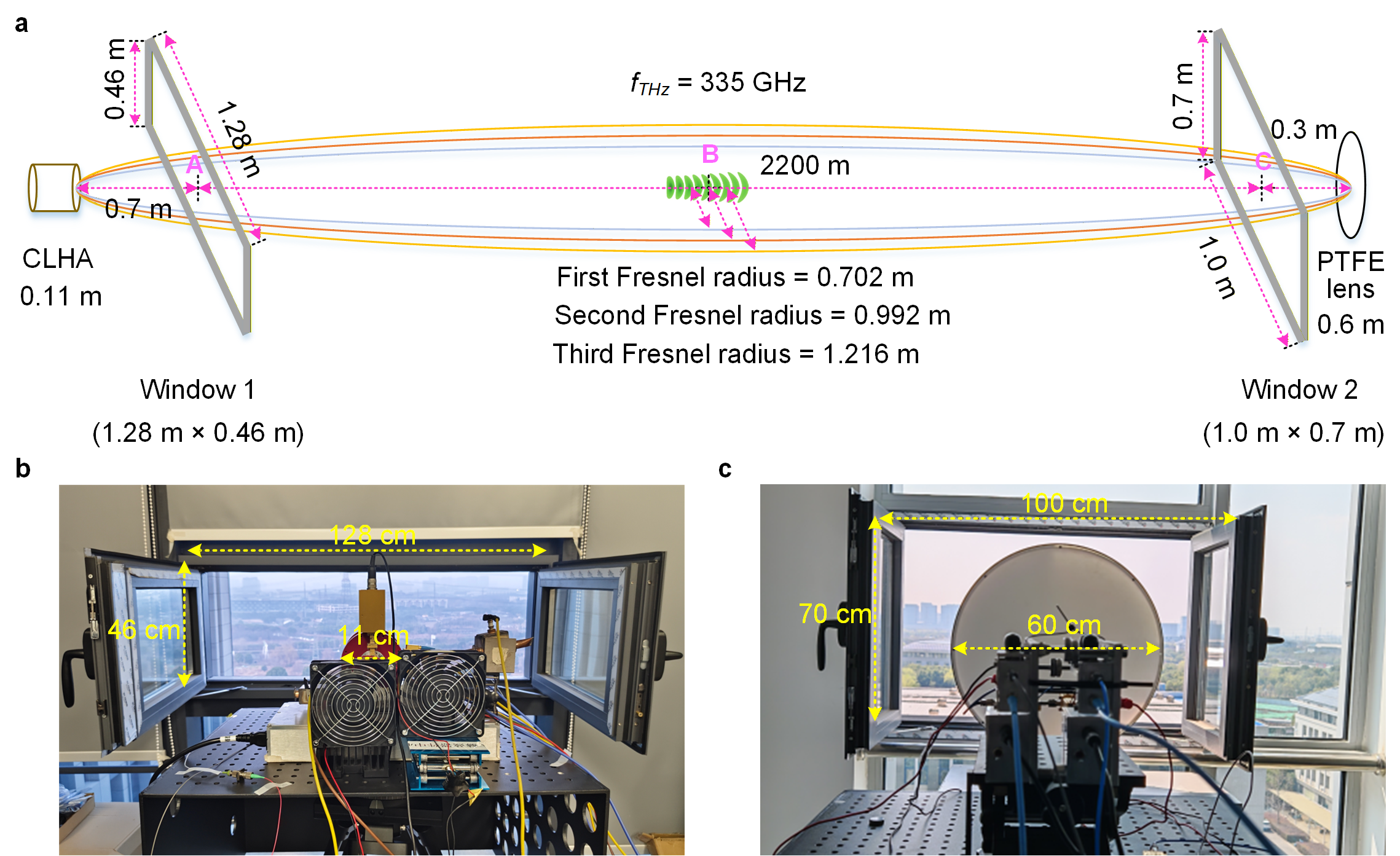


**Fig. S5 | Far-field region and Fresnel zone radius analysis. a,** Schematic of the wireless link between the transmitting CLHA and the receiving PTFE lens. **b,** Photo of the THz transmitter. **c,** Photo of the THz receiver. CLHA, cylindrical lens horn antenna; PTFE, polytetrafluoroethylene.

The carrier frequency in this system is set to *f*_THz_ = 335 GHz, and the diameters of the CLHA and the PTFE lens are around 11 and 60 cm, respectively. As the aperture of the horn antenna at the receiving end is much smaller than that of the PTFE lens, the far-field region is mainly determined by the latter. For CLHA, the Fraunhofer boundary *d*_tx_ = 2 × 0.11^2^ × 335 × 10^9^ / (3 × 10^8^) = 27.02 m, whereas the Fraunhofer boundary *d*_rx_ = 2 × 0.6^2^ × 335 × 10^9^ / (3 × 10^8^) = 804 m for the PTFE lens. As both Fraunhofer distances of the transmitting CLHA and the receiving PTFE lens are smaller than our wireless transmission distance of 2.2 km, our 2.2 km THz communication system operates in the far-field mode.

Another important consideration is whether the windows obstruct the Fresnel zone at their current size, as such obstruction can significantly affect the system’s performance. The radius of the *k*th Fresnel zone can be calculated as follows^7^:

$r_{k}=\sqrt{\frac{kd_{1}d_{2}}{d_{1}+d_{2}}}$ (S2)

where *k =* 1, 2, and 3 represent the first, second, and third Fresnel zones, respectively. *d*_1_ and *d*_2_ are the distances from the observation point to the CLHA and PTFE lens, respectively.

Using Eq. (S2), we can calculate the first to third Fresnel radii at window 1 (point A), the midpoint (point B), and window 2 (point C), respectively. The results are shown in Table S1. The maximum Fresnel radius at the two window ends is 4.3 cm—much smaller than the length and width of the windows. Therefore, neither window obstructs the Fresnel zone in our 2.2 km THz link. Moreover, the midpoint has a maximum Fresnel radius of about 1.2 m, and its Fresnel zone is not obstructed by buildings between the links.

Table S1 | Radius of the *k*th Fresnel zone at three different observation points

| Observation point | Radius | | |
| --- | --- | --- | --- |
|  | First Fresnel zone (m) | Second Fresnel zone (m) | Third Fresnel zone (m) |
| A  (*d*_1_ = 0.7, *d*_2_ = 2200.3) | 0.025 | 0.035 | 0.043 |
| B  (*d*_1_ = *d*_2_ = 1100.5) | 0.702 | 0.992 | 1.216 |
| C  (*d*_1_ = 2200.7, *d*_2_ = 0.3) | 0.016 | 0.023 | 0.028 |

**S2.2 THz power budget**

In the far-field operating case, according to Friis’ equation^8^, the free space path loss (FSPL) can be given as

$FSPL(dB)=20\times\log_{10}\left(\frac{4\pi Lf}{c}\right)$ (S3)

where *L* is the wireless transmission distance, *f* is the THz carrier frequency, and *c* denotes the speed of light in vacuum. In our experiment, after transmitting a 335 GHz THz signal over 2.2 km, the corresponding FSPL is about 149.8 dB. In addition, considering an environment with a temperature of 15 ℃, relative humidity of 40%, and standard atmospheric pressure, the atmospheric absorption loss (hereafter, *L*_at_) over 2.2 km for the 335 GHz THz wave is about 15.8 dB.

Table S2 shows the power budget of our 2.2 km THz wireless link. Considering that the average output power of TWTA is *P*_T_ = 30 dBm, the gain of integrated CLHA at the transmitter is *G*_T_ = 48.5 dBi, whereas the total gain of the PTFE lens and horn antenna at the receiver is *G*_R_ = 58 dBi. Consequently, the received power of the THz signal after the antenna can be calculated as *P*_R_ *=* *P*_T_ + *G*_T_ – *FSPL* − *L*_at_ + *G*_R_ = −29.1 dBm. The actual measured power is less than this value because it includes some additional losses, such as connection loss, energy loss due to the insufficient receiving area of the lens at the receiving end, and so on.

Table S2 | Power budget of the 2.2 km THz wireless link

| Parameter | Typical/nominal value | Remark |
| --- | --- | --- |
| *P*_T_ | 30 dBm | Average power output by TWTA |
| *G*_T_ | 48.5 dBi | Gain of CLHA at the Tx side |
| FSPL | 149.8 dB | Loss of the 2.2 km wireless link at 335 GHz |
| *L*_at_ | 15.8 dB | Atmospheric absorption loss of the 2.2 km wireless link at 335 GHz |
| *G*_R_ | 58 dBi | Total gain of the PTFE lens + horn antenna at the Rx side |
| *P*_R_ | −29.1 dBm | Power of the received THz signal after the antenna |

Although 335 GHz is chosen for the long-range THz transmission demonstration here, the techniques used in this article are also suitable for other THz frequency bands. One the one hand, the photonics-assisted THz generation approach is inherently frequency-agile by using tunable laser sources, whilst the diversity reception technique is suitable for a wide range of THz frequencies. On the other hand, just like traditional all-solid-state electronic components, the electrical THz amplification module also has the possibility of expanding to other frequency bands by redesigning the vacuum-electronics-based TWTA.

In addition to 335 GHz case, we further predict the transmission performance of other carrier frequencies, taking 220 GHz and 420 GHz as examples. The TWTA designed for 220 GHz can enable an output peak power of 43 dBm^9^, whilst for 420 GHz, we roughly predict the peak power to be around 30 dBm based on our simulation results. Referring to the 335 GHz TWTA case, we uniformly use a 6 dB reduction in peak power as its average power. Assuming that the receiving sensitivity and device gain of the link are consistent with the 335 GHz TWTA for simplicity, then we can calculate that the maximum link loss for 220 GHz and 420 GHz are 172.6 dB and 159.6 dB, respectively. Then the predicted wireless transmission distances for these two THz band correspond to 8280 m and 1030 m according to Eq. (S3), respectively.

Table S3 | Transmission performance prediction for other carrier frequencies

| THz carrier frequency | Output peak/average power of TWTA | Maximum link loss | Predicted wireless distance |
| --- | --- | --- | --- |
| 220 GHz | 43 dBm / 37 dBm | 165.6 + 7 = 172.6 dB | 8280 m |
| 335 GHz | 36 dBm / 30 dBm | 149.8 + 15.8 = 165.6 dB | 2200 m |
| 420 GHz | 30 dBm / 24 dBm | 165.6 − 6 = 159.6 dB | 1030 m |

**S3.** **Theoretical model for a single-emission and double-reception** **diversity scheme**

A spatial diversity scheme based on single emission and double reception (Fig. S6a) is used to improve the receiving signal-to-noise ratio (SNR) and sensitivity. Notably, this structure differs from the common single-emission and double-reception scheme in traditional wireless communication systems, which builds two independent and entire wireless transmission channels. Instead, we share the main long-range THz wireless transmission link with significant improvement in operability, while two independent channels are built after the PTFE lens using two adjacent THz receivers. Fig. S6b shows the double THz receivers used in our experiment. As the noises introduced by the two independent THz receiving channels (including frequency multiplication, downconversion, and amplification) are uncorrelated with each other, the SNR can be improved after a maximum ratio combining (MRC) operation based on dual-channel information redundancy^10^.

According to the description in **Methods: Theoretical model of SNR gain for** **a single-emission and double-reception** **diversity scheme** in the main manuscript, we know the following two basic conditions: 1) the mean values of the signal and the noise are zero—that is, *E*[*s*] = *E*[*n*_0_] = *E*[*n*_1_] = *E*[*n*_2_] = 0, where *E*[*x*] stands for the expectation of *x*. 2) All three noises are uncorrelated with each other—that is, *E*[*n*_0_*n*_1_] = *E*[*n*_0_*n*_2_] = *E*[*n*_1_*n*_2_] = 0.


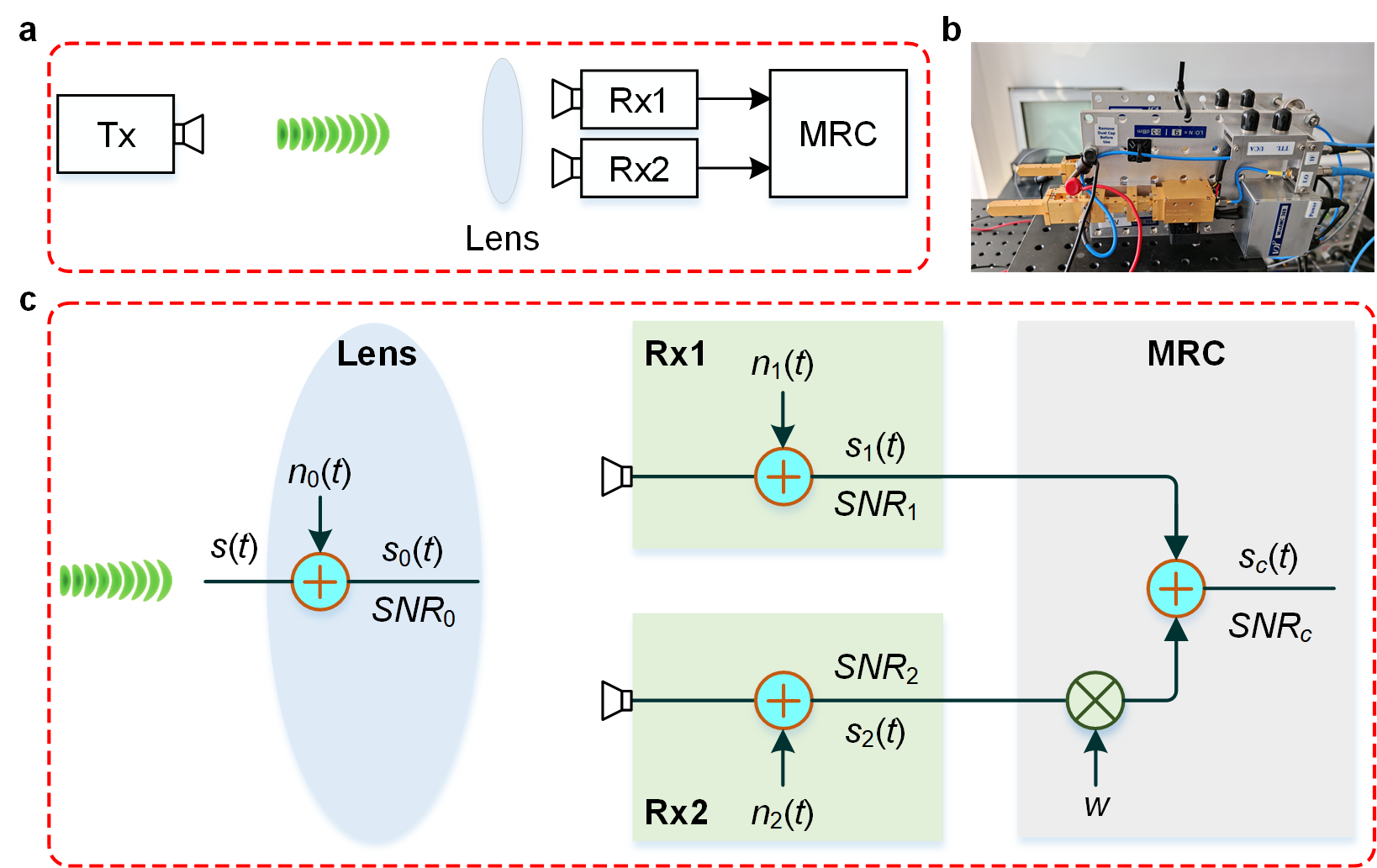


**Fig. S6 | Single-emission and double-reception diversity scheme. a,** Concept of the single-emission and double-reception diversity scheme. **b,** Photo of the double THz receivers. **c,** Theoretical model for the single-emission and double-reception diversity scheme. Tx, transmitter; Rx, receiver; MRC, maximum ratio combining.

Fig. S6c shows the theoretical model of the SNR gain for the single-emission and double-reception diversity scheme. Given the abovementioned two conditions, we can obtain the following expressions:

$s_{0}(t)=s(t)+n_{0}(t)$ (S4)

$SNR_{0}=E\left[s^{2}\right]/E\left[n_{0}^{2}\right]$ (S5)

$s_{i}(t)=s(t)+n_{0}(t)+n_{i}(t)$ (S6)

$SNR_{i}=E\left[s^{2}\right]/E\left[(n_{0}+n_{i})^{2}\right]=E\left[s^{2}\right]/\left(E\left[n_{0}^{2}\right]+E\left[n_{i}^{2}\right]\right)$ (S7)

where *i* = 1 or 2 represents the index number of the receiving channel.

According to Eq. (S5) and (S7), the noise power can be represented by the SNR:

$E\left[n_{0}^{2}\right]=E\left[s^{2}\right]SNR_{0}^{-1}$ (S8)

$E\left[n_{i}^{2}\right]=E\left[s^{2}\right]\left(SNR_{i}^{-1}-SNR_{0}^{-1}\right)$ (S9)

After performing MRC processing for the two receiving signals with a weight factor *w* (*w*ℝ^+^), the resultant signal and SNR can be expressed as

$s_{c}(t)=s_{1}(t)+ws_{2}(t)=(1+w)s(t)+(1+w)n_{0}(t)+n_{1}(t)+wn_{2}(t)$ (S10)

$\begin{matrix} SNR_{c}(w)=\frac{E\left[(1+w)^{2}s^{2}\right]}{E\left[\left((1+w)n_{0}+n_{1}+wn_{2}\right)^{2}\right]} \\ =\frac{(1+w)^{2}E\left[s^{2}\right]}{(1+w)^{2}E\left[n_{0}^{2}\right]+E\left[n_{1}^{2}\right]+w^{2}E\left[n_{2}^{2}\right]} \end{matrix}$ (S11)

Letting *F*(*w*) = 1/*SNR_c_*(*w*), we obtain

$F(w)=\frac{E\left[n_{0}^{2}\right]}{E\left[s^{2}\right]}+\frac{1}{(1+w)^{2}}\frac{E\left[n_{1}^{2}\right]}{E\left[s^{2}\right]}+\left(\frac{w}{1+w}\right)^{2}\frac{E\left[n_{2}^{2}\right]}{E\left[s^{2}\right]}$ (S12)

To maximize *SNR_c_*(*w*), we need to minimize *F*(*w*). By solving for *dF*(*w*)/*dw* = 0, we can obtain the optimal weight coefficient *w*_opt_ *=* *E*[*n*_1_^2^]/*E*[*n*_2_^2^]. In this case, 1/(1 + *w*) = *E*[*n*_2_^2^]/(*E*[*n*_1_^2^] + *E*[*n*_2_^2^]), and *w*/(1 + *w*) = *E*[*n*_1_^2^]/(*E*[*n*_1_^2^] + *E*[*n*_2_^2^]). Then, the minimum *F*(*w*) is given as

$\begin{matrix} F\left|_{w=w_{opt}}\right.=\frac{E\left[n_{0}^{2}\right]}{E\left[s^{2}\right]}+\left(\frac{E\left[n_{2}^{2}\right]}{E\left[n_{1}^{2}\right]+E\left[n_{2}^{2}\right]}\right)^{2}\frac{E\left[n_{1}^{2}\right]}{E\left[s^{2}\right]}+\left(\frac{E\left[n_{1}^{2}\right]}{E\left[n_{1}^{2}\right]+E\left[n_{2}^{2}\right]}\right)^{2}\frac{E\left[n_{2}^{2}\right]}{E\left[s^{2}\right]} \\ =\frac{1}{E\left[s^{2}\right]}\left\{E\left[n_{0}^{2}\right]+\frac{E\left[n_{1}^{2}\right]E\left[n_{2}^{2}\right]}{E\left[n_{1}^{2}\right]+E\left[n_{2}^{2}\right]}\right\} \end{matrix}$ (S13)

According to Eq. (S9), the second term of Eq. (S13) can be expanded as follows:

$\begin{matrix} \frac{E\left[n_{1}^{2}\right]E\left[n_{2}^{2}\right]}{E\left[n_{1}^{2}\right]+E\left[n_{2}^{2}\right]}=\frac{E\left[s^{2}\right]\left(\frac{1}{SNR_{1}}-\frac{1}{SNR_{0}}\right)\cdot E\left[s^{2}\right]\left(\frac{1}{SNR_{2}}-\frac{1}{SNR_{0}}\right)}{E\left[s^{2}\right]\left(\frac{1}{SNR_{1}}-\frac{1}{SNR_{0}}\right)+E\left[s^{2}\right]\left(\frac{1}{SNR_{2}}-\frac{1}{SNR_{0}}\right)} \\ =E\left[s^{2}\right]\cdot\frac{\left(\frac{SNR_{0}-SNR_{1}}{SNR_{0}SNR_{1}}\right)\cdot\left(\frac{SNR_{0}-SNR_{2}}{SNR_{0}SNR_{2}}\right)}{\left(\frac{1}{SNR_{1}}+\frac{1}{SNR_{2}}-\frac{2}{SNR_{0}}\right)} \\ =E\left[s^{2}\right]\cdot\frac{\left(\frac{\left(SNR_{0}-SNR_{1}\right)\left(SNR_{0}-SNR_{2}\right)}{SNR_{0}^{2}SNR_{1}SNR_{2}}\right)}{\left(\frac{SNR_{0}SNR_{2}+SNR_{0}SNR_{1}-2SNR_{1}SNR_{2}}{SNR_{0}SNR_{1}SNR_{2}}\right)} \\ =E\left[s^{2}\right]\cdot\frac{\left(SNR_{0}-SNR_{1}\right)\left(SNR_{0}-SNR_{2}\right)}{SNR_{0}^{2}\left(SNR_{1}+SNR_{2}\right)-2SNR_{0}SNR_{1}SNR_{2}} \end{matrix}$ (S14)

According to Eqs. (S8) and (S14), Eq. (S13) can be rewritten as

$\begin{matrix} F\left|_{w=w_{opt}}\right.=\frac{1}{E\left[s^{2}\right]}\left\{\frac{E\left[s^{2}\right]}{SNR_{0}}+E\left[s^{2}\right]\cdot\frac{\left(SNR_{0}-SNR_{1}\right)\left(SNR_{0}-SNR_{2}\right)}{SNR_{0}^{2}\left(SNR_{1}+SNR_{2}\right)-2SNR_{0}SNR_{1}SNR_{2}}\right\} \\ =\frac{SNR_{0}\left(SNR_{1}+SNR_{2}\right)-2SNR_{1}SNR_{2}+\left(SNR_{0}-SNR_{1}\right)\left(SNR_{0}-SNR_{2}\right)}{SNR_{0}^{2}\left(SNR_{1}+SNR_{2}\right)-2SNR_{0}SNR_{1}SNR_{2}} \\ =\frac{SNR_{0}^{2}-SNR_{1}SNR_{2}}{SNR_{0}^{2}\left(SNR_{1}+SNR_{2}\right)-2SNR_{0}SNR_{1}SNR_{2}} \end{matrix}$ (S15)

Then, we can obtain the maximum *SNR_c_*:

$\begin{matrix} SNR_{c}\left|_{w=w_{opt}}\right.=F^{-1}\left|_{w=w_{opt}}\right. \\ =\frac{SNR_{0}^{2}\left(SNR_{1}+SNR_{2}\right)-2SNR_{0}SNR_{1}SNR_{2}}{SNR_{0}^{2}-SNR_{1}SNR_{2}} \end{matrix}$ (S16)

**S4. Supplementary information for long-range THz wireless communication**

**S4.1.** **Detailed flowchart for the LSTM–ANN equalizer**

Traditional MRC processing explicitly calculates the weight of each branch on the basis of the transmission characteristics of the channel. Thus, its performance is significantly affected by the time-varying characteristics of long-range THz wireless links. Here, we enable a long-short-term memory–artificial neural network (LSTM–ANN) equalizer to implicitly learn an optimal merge strategy using a data-driven approach. This LSTM–ANN equalizer effectively handles time sequences and overcomes time-varying characteristics given its time memory capability.


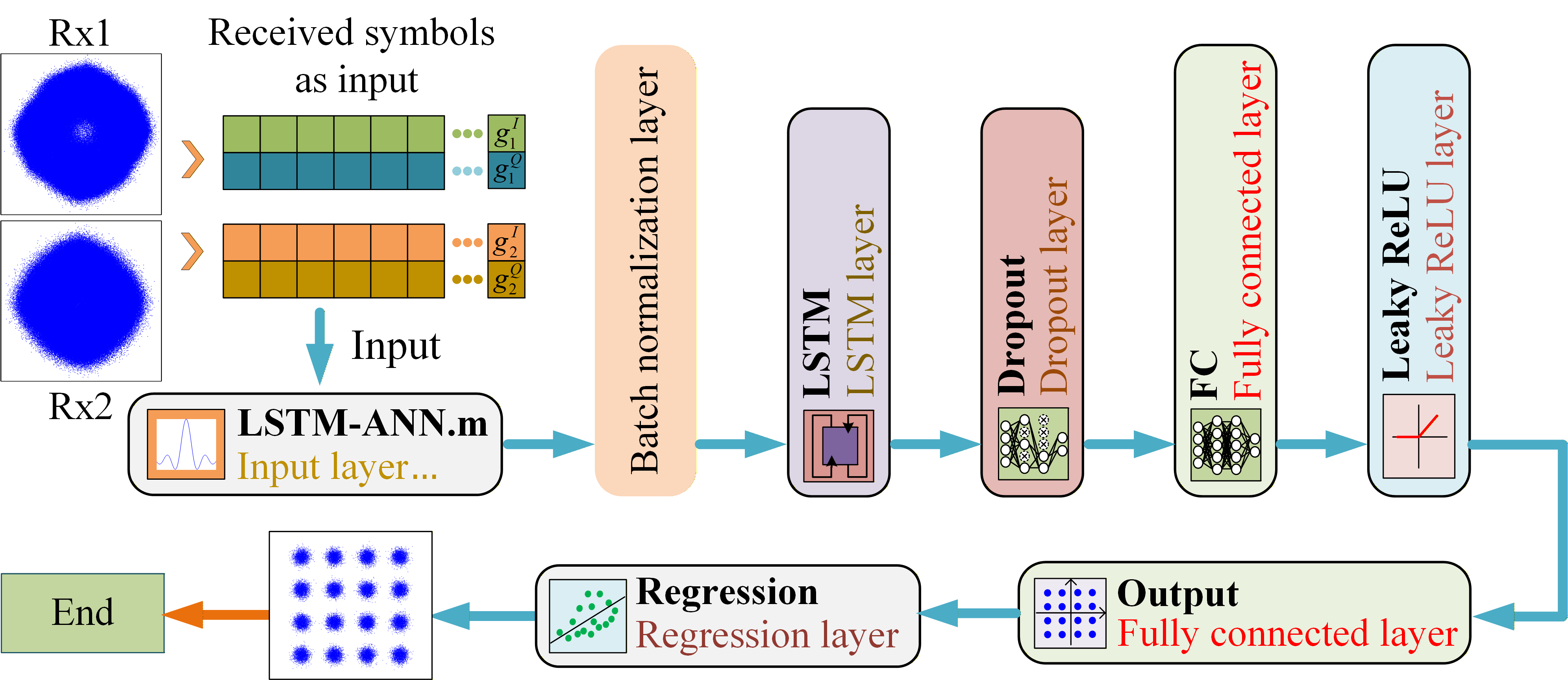


**Fig. S7 | Flowchart of the proposed LSTM–ANN equalizer.**

The detailed structure of the LSTM–ANN equalizer is shown in Fig. S7. It mainly consists of a neural network with eight layers—an input layer, a batch normalization layer, an LSTM layer, a dropout layer, two fully connected (FC) ANN layers, a leaky rectified linear unit (ReLU) layer, and a regression layer. The LSTM–ANN input layer with a dimension of 4 contains time sequences of real and imaginary parts of the received signals from two channels. The batch normalization layer normalizes the input to accelerate network training and improve stability by reducing the impact of amplitude differences among different signals at the same time. Subsequently, the LSTM layer (with 64 hidden units) designs and trains the network to operate in sequence mode according to the characteristics of the input sequence, producing predicted outputs of the same size as the input sequence. This LSTM layer captures intersymbol interference and channel memory effects and learns channel time-varying characteristics through gating mechanisms. To prevent overfitting, lightweight regularization is performed by randomly discarding 10% of the neurons in the dropout layer. Thereafter, the first FC-ANN layer with 30 neurons and the leaky ReLU layer further achieve feature compression and nonlinear transformation to learn more complex merging and equalization mappings. Then, the second FC-ANN layer (with a dimension of 2) is selected as the output layer to generate the eventual predicted real and imaginary parts of the symbol sequences. Finally, the training loss is calculated using the mean square error at the regression layer.

After multiple iterations, the LSTM–ANN network obtains long-term dependencies through supervised learning methods while achieving weight-adaptive implicit MRC and end-to-end channel equalization of a 16-ary quadrature amplitude modulation (16QAM) signal. Although our proposed LSTM–ANN network performs well within the specific operational scope for which it was designed and trained, its generalization capability is indeed limited by factors such as channel environment, modulation format, and system architecture. The change in the above conditions requires a redesign and training of the network model.

**S4.2. Performance under different times of the day and humidity conditions**

This long-range THz outdoor experiment was conducted in Nanjing, China under clear weather in February. The environmental condition is as follows: 10–18 ℃ temperature, 36%–50% relative humidity, and standard atmospheric pressure. Based on the proposed hybrid photonic–electronic synergy scheme, the SNR performance under different times of the day and humidity conditions is also shown in Fig. S8. It can be seen that the SNR performance is not exactly on the same scale as humidity. This is because, in addition to the performance fluctuations of the link itself, the transmission path loss is determined by both temperature and humidity, while the temperature also varies at different times. This THz link demonstrates good stability, showing less than 1 dB SNR fluctuations within 7 hours.


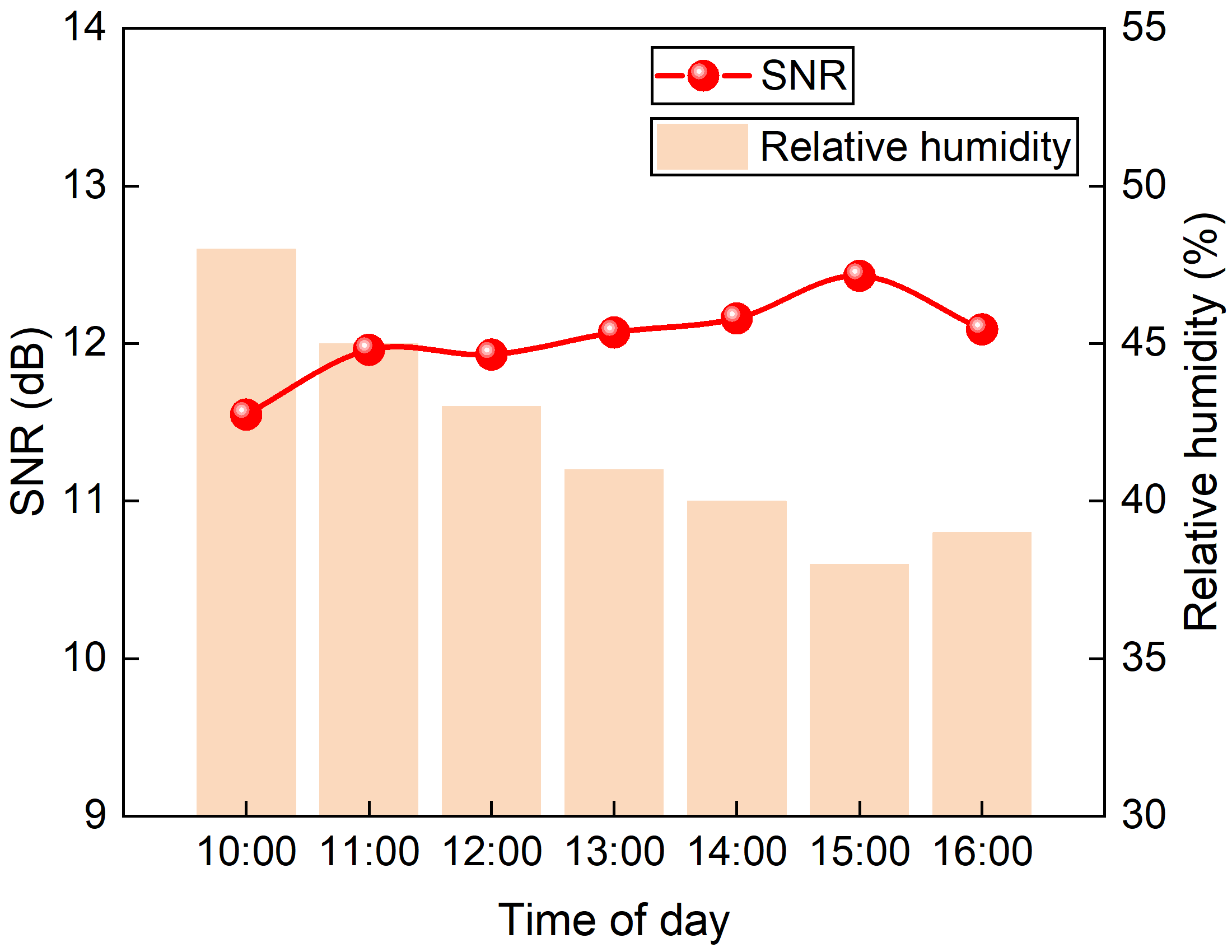


**Fig. S8 | SNR transmission performance under different times of day and humidity conditions.**

**S4.3. Real-time demonstration of 5G new radio (NR) and live video transmission**

In this section, using the 2.2 km long-range THz wireless link, we further demonstrate the real-time transmission of 5G NR signals and high-definition (HD) live video. As the above demonstrations both involve real-time signal processing, a single THz receiver is used at the receiving end for simplicity. Figs. S9a and S9b show the corresponding transmitting and receiving ends, respectively. At the transmitting end, a standard 5G NR signal with a center frequency of 2.6 GHz is generated in real time by a vector signal generator (Ceyear, 1466L-V). For the video stream, as shown in Fig. S9a, a real-time uncompressed 1.485 Gbps HD video with a pixel resolution of 1920 × 1080 is captured by one camera and output via its high-definition multimedia interface (HDMI) port. This HDMI video stream is then converted to a serial digital interface (SDI) data stream with non-return-to-zero (NRZ) coding by one HDMI-SDI converter. To enhance the resistance of the real-time demonstration system to polarization disturbances, two external cavity laser diodes (LD1 and LD2) with a frequency interval of 335 GHz are directly coupled through a polarization-maintaining optical coupler. Then, the 2.6 GHz 5G NR or video NRZ baseband (BB) signal is modulated onto two lightwaves via a simple intensity modulator (IM). This configuration, combined with a receiver-side envelope detector (ED), ensures robustness against the frequency offset and phase noise resulting from the two free-running LDs. Subsequently, the two modulated lightwaves are converted to a 335 GHz THz signal after amplification. Similar to the previous operation, the generated 335 GHz THz signal is first amplified by a continuous-wave TWTA and is then emitted into the air for long-range wireless transmission over 2.2 km.


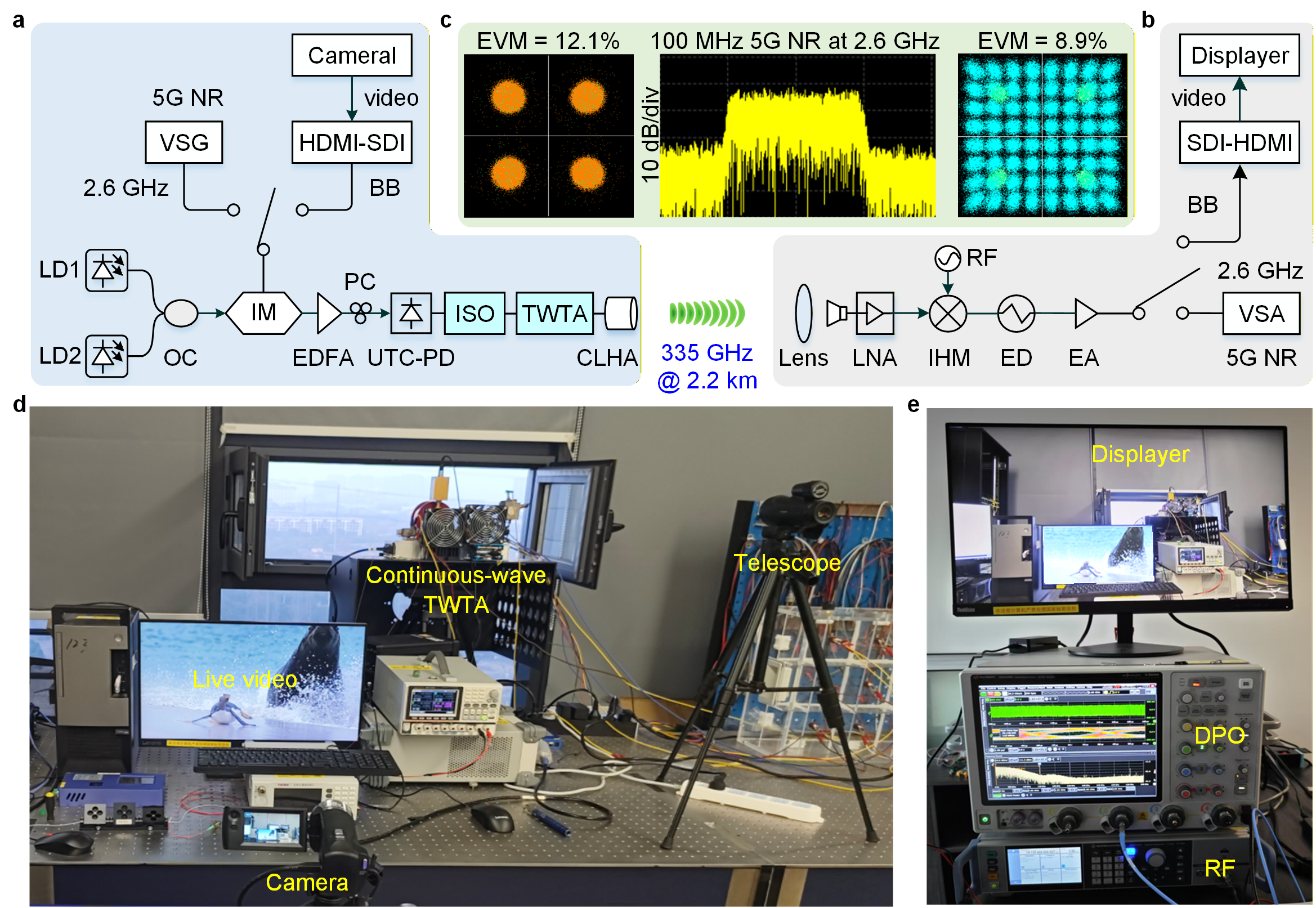


**Fig. S9 | Real-time demonstration of 5G NR and live video transmission. a,** Experimental setup of the 5G NR and video transmitting end. **b,** Experimental setup of the 5G NR and video receiving end. **c,** Transmission performance and spectrum of the 100-MHz 5G NR signal at 2.6 GHz. **d,** Photo of the live video transmitting end. **e,** Photo of the video receiving end. NR, new radio; VSG, vector signal generator; HDMI, high-definition multimedia interface; SDI, serial digital interface; BB, baseband; IM, intensity modulator; EDFA, erbium-doped fiber amplifier; PC, polarization controller; UTC-PD, uni-traveling carrier photodiode; ISO, isolator; IHM, integrated harmonic mixer; ED: envelope detector, RF, radio frequency; VSA, vector signal analyzer; DSO, digital storage oscilloscope.

At the receiving end, as shown in Fig. S9b, the received 335 GHz THz signal is focused by a PTFE lens and amplified by a THz low-noise amplifier. Because of the absence of available THz ED in the 335 GHz frequency band, we implement a hybrid downconversion method through frequency mixing followed by envelope detection. Specifically, the 335 GHz THz signal is first downconverted to 4.7 GHz IF using an integrated harmonic mixer (IHM). This IF signal is then further detected using an ED (ADI, ADL6012) with an operation frequency of 2–67 GHz to extract the envelope information, thereby recovering the 2.6 GHz 5G NR or video NRZ BB signal. Thereafter, the 2.6 GHz 5G NR signal is directly fed into a vector signal analyzer (Ceyear, 4082L) for performance evaluation. Meanwhile, an SDI-HDMI converter is used to convert the video NRZ BB signal into an HDMI video stream, which is then sent to a video displayer for real-time display.

Fig. S9c shows the received electrical spectrum of the 100 MHz 5G NR signal with a center frequency of 2.6 GHz. The SNR is around 20 dB. The measured error vector amplitudes (EVMs) for quadrature phase shift keying (QPSK) and 64-ary quadrature amplitude modulation (64QAM) signals correspond to 12.1% and 8.9%, respectively. Both values meet the 3GPP-TS38.141 technical specifications, where the EVM thresholds for QPSK and 64QAM are defined as 18.5% and 9%, respectively. As the 2.6 GHz 5G NR signal occupies an actual bandwidth above 5.2 GHz after IM modulation, higher-order modulation formats are constrained by the operation bandwidth of the TWTA in this demonstration.

Figs. S9d and S9e show photos of the live video transmitting and receiving ends, respectively. The HD video is successfully demonstrated over a 2.2 km long-range THz wireless transmission at a carrier frequency of 335 GHz. At the receiving end, the DPO also presents a time-domain waveform, eye diagram, and frequency-domain spectrum of the received 1.485 Gbps video NRZ BB signal. Additionally, the robustness and stability of this transmission system are further proven by 2 hours of uninterrupted real-time live video.

**Supplementary references**

1. Paoloni, C. et al. Technology for D-band/G-band ultra capacity layer. Proceedings of 2019 European Conference on Networks and Communications (EuCNC). Valencia, Spain: IEEE, 2019, 209-213.
2. Yoshida, M. et al. Development activity of terahertz amplifiers with FWG-TWTs. Proceedings of 2016 IEEE International Vacuum Electronics Conference (IVEC). Monterey, CA, USA: IEEE, 2016, 1-2.
3. Cutler, C. C. & Hines, M. E. Thermal velocity effects in electron guns. *Proceedings of the IRE* **43**, 307-315 (1955).
4. Wu, H. et al. Generation and stable transmission of miniature electron beams for 0.65-THz traveling wave tubes. *IEEE Transactions* *Electron Devices* **71**, 2116-2121 (2024).
5. Gilmour, A. S. Principles of traveling wave tubes. (Boston, MA: Artech House, 1995).
6. Selvan, K. T. & Janaswamy, R. Fraunhofer and Fresnel Distances: unified derivation for aperture antennas. *IEEE Antennas and Propagation Magazine* **59**, 12-15 (2017).
7. Li, W. et al. Photonic terahertz wireless communication: towards the goal of high-speed kilometer-level transmission. *Journal of Lightwave Technology* **42**, 1159-1172 (2024).
8. Friis, H. T. A note on a simple transmission formula. *Proceedings of the IRE* **34**, 254-256 (1946).
9. Pan, P. et al. A G-band traveling wave tube with 20 W continuous wave output power. *IEEE Electron Device Letters* **41**, 1833-1836 (2020).
10. Horst, Y. et al. Dual sideband receiver for radio-over-fiber. *Optics Express* **32**, 4305-4316 (2024).
